# Expression, Clinical Significance, and Functional Prediction of *MNX1* in Breast Cancer

**DOI:** 10.1016/j.omtn.2018.09.014

**Published:** 2018-09-27

**Authors:** Tian Tian, Meng Wang, Yuyao Zhu, Wenge Zhu, Tielin Yang, Hongtao Li, Shuai Lin, Cong Dai, Yujiao Deng, Dingli Song, Na Li, Zhen Zhai, Zhi-Jun Dai

**Affiliations:** 1 Department of Breast Surgery, Guangzhou Women and Children’s Medical Center, Guangzhou Medical University, Guangzhou 510623, Guangdong, China; 2 Department of Oncology, The Second Affiliated Hospital of Xi’an Jiaotong University, Xi’an 710004, China; 3 Department of Biochemistry and Molecular Medicine, The George Washington University Medical School, Washington, DC 20052, USA; 4 School of Life Science and Technology, Xi’an Jiaotong University, Xi’an 710049, China; 5 Department of Breast, Head and Neck Surgery, Affiliated Tumor Hospital of Xinjiang Medical University, Urumchi 830000, China

**Keywords:** motor neuron and pancreas homeobox 1, *MNX1*, breast cancer, clinical, expression, prognosis

## Abstract

Motor neuron and pancreas homeobox 1 (*MNX1*) is a key developmental gene. Previous studies found that it was upregulated in several tumors, but its role in breast cancer (BC) remains unclear. In order to have a better understanding of this gene in BC, we examined the expression of *MNX1* in BC tissues and normal breast tissues by qRT-PCR and by analyzing data from The Cancer Genome Atlas (TCGA) database. We also assessed the association of *MNX1* expression with BC clinicopathological features and investigated the impact of *MNX1* on BC survival. Potential molecular function of *MNX1* was predicted through protein-protein interactions and functional enrichment. The results showed that the expression of *MNX1* was significantly increased in BC tissues, especially in the HER2-positive subtype, and *MNX1* expression was associated with several clinical characteristics, including menopause status, receptor status, subtypes, tumor size, lymph node metastasis, and race. In addition, patients with higher *MNX1* expression had poorer survival. Enrichment analysis suggested that *MNX1* is probably involved in biological processes and pathways related to nuclear division, cell cycle, and p53 signaling. In conclusion, our study suggests that *MNX1* may act as a tumor promoter in BC. We hope these findings will draw more attention to *MNX1* in future cancer studies.

## Introduction

Breast cancer (BC) is the most common cancer type and the leading cause of global cancer death among females.^1^ Although cancer treatment has reached the era of precision medicine, the precise treatment of BC remains a challenge, because no effective targets have been found except for a few validated biomarkers, such as estrogen receptor (ER), HER2, PIK3CA, and AKT1. Currently, the application of targeted therapy in BC is limited.^2^ Therefore, the study of molecular mechanisms underlying BC and novel oncogenic drivers is important, which may lead to the identification of potential therapeutic targets for BC.

*MNX1*, located on human chromosome 7q36.3, belongs to the family of homeobox genes. It encodes a nuclear protein named motor neuron and pancreas homeobox 1 (MNX1), also known as HLXB9 or HB9, which is a transcription factor.^3^ *MNX1* is a key developmental gene that is normally expressed in neurons as well as pancreatic and lymphoid cells. It is involved in both motor neuronal differentiation and pancreatic beta cell development.^4^, ^5^, ^6^ Defects in this gene result in hereditary sacral agenesis, which is also called Currarino syndrome.^7^, ^8^ The function of *MNX1* in cancer biology has not been clarified. However, the expression of *MNX1* has been reported to be upregulated in several tumors, including prostate cancer, hepatocellular carcinoma (HCC), acute myeloid leukemia (AML), and neuroblastoma.^3^, ^9^, ^10^, ^11^ Furthermore, it has been demonstrated to be oncogenic in prostate cancer and insulinoma.^12^, ^13^

Although evidence has shown that *MNX1* may play a role in tumorigenesis, its expression and function in BC is still unclear by far. This gene had hardly been studied in BC before. Only Neufing et al.^14^ have reported that the percentage of nuclei expressing of MNX1 is increased in breast carcinoma. However, the intensity of nuclear staining is decreased progressively with increasing tumor grade. In order to have a better understanding of *MNX1*in BC, we investigated its expression profile and clinical significance in BC and the impact of its expression on BC survival while also exploring the potential molecular function through bioinformatic analysis and experimental method.

## Results

### *MNX1* Expression Is Upregulated in BC Tissues

**Figure 1:**
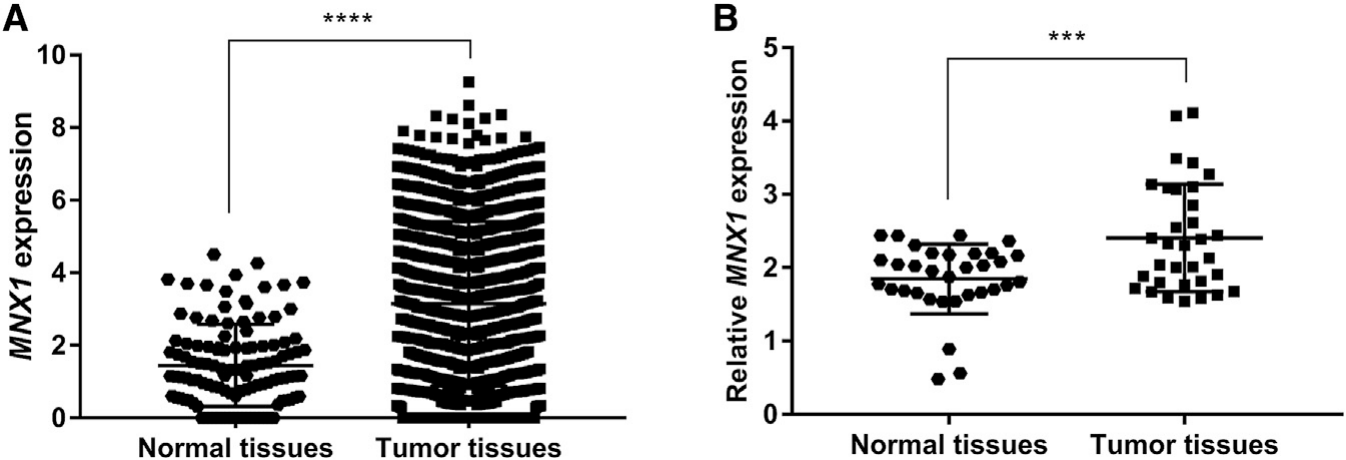
The Expression of *MNX1* Was Significantly Increased in BC Tissues compared with Normal Breast Tissues (A) Analysis of *MNX1* expression profile using data from TCGA. (B) *MNX1* expression was detected by qRT-RCR in 33 pairs of BC tissues and normal breast tissues. ***p < 0.0001 and ****p = 0.0006.

**Figure 2:**
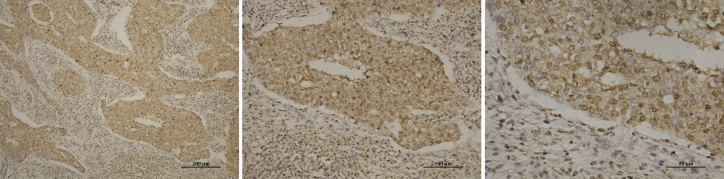
Representative Microscopy Images of BC Sections Images are at (left) 10×, (middle) 20×, and (right) 40× magnifications, respectively, under the same field.

We explored the expression profile of *MNX1* in BC tissues using The Cancer Genome Atlas (TCGA) dataset. There were 1,218 samples in the dataset, including 1,104 BC tissues and 114 normal breast tissues. The results indicated that, compared with the normal breast tissues, the level of *MNX1* was significantly increased in BC tissues (p < 0.0001; Figure 1A). Similarly, qRT-PCR results also revealed that *MNX1* expression was significantly upregulated in BC tissues (p = 0.0006; Figure 1B). Immunohistochemistry showed that MNX1 protein was mainly expressed in the nucleus of BC cells (Figure 2).

### *MNX1* Expression Is Associated with BC Clinicopathology and Survival

**Figure 3:**
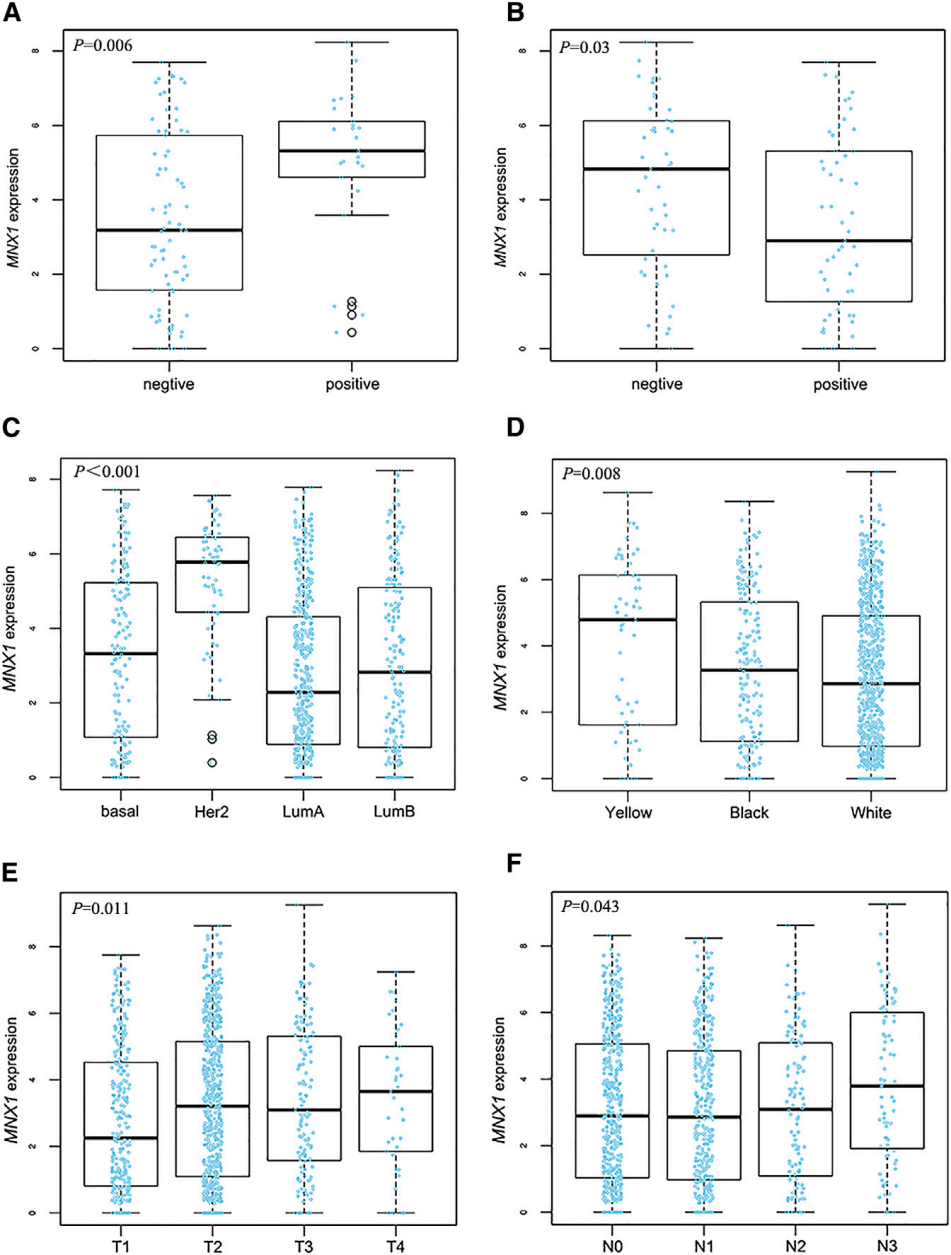
The Relationship between *MNX1* Expression and BC Clinical Features: Analyses of Data from TCGA (A) *MNX1* expression and Her2 status. (B) *MNX1* expression and PR status. (C) *MNX1* expression in different subtypes. (D) *MNX1* expression in different races. (E) *MNX1* expression and T stage. (F) *MNX1* expression and N stage.

The relationship between *MNX1* expression and BC clinicopathological features was assessed in 60 BC patients. The median *MNX1* expression of all BC tissues was used as the cutoff value to divide BC patients into two groups. As shown in Table 1, *MNX1* expression was related to menopause status and Her2 expression. We subsequently analyzed the data from TCGA. We observed that the level of *MNX1* was higher in Her2-positive and progesterone receptor (PR)-negative tissues (Figures 3A and 3B). Consistently, *MNX1* was significantly upregulated in Her2-positive BC compared with that in the other three subtypes of BC (Figure 3C). In addition, *MNX1* level differs with race. The expression in Asians was the highest (Figure 3D). Moreover, patients with advanced tumor (T) and lymph node (N) stages tend to have an increased *MNX1* level (Figures 3E and 3F).

**Table 1:** Relationship between *MNX1* Expression and Clinicopathological Features of Breast Cancer Patients

Clinicopathological Parameters	*MNX1* Expression		OR	p Value
	High (30)	Low (30)		
**Age (Years)**				

55	14	10	1.750	0.292
≤55	16	20		
**Menopause Status**				

Postmenopausal	22	14	3.143	0.035∗
Premenopausal	8	16		
**Tumor Size**				

>2 cm	18	13	1.962	0.196
≤2 cm	12	17		
**Lymph Node Metastasis**				

Positive	21	16	2.042	0.184
Negative	9	14		
**TNM Stage**				

III–IV	6	3	2.250	0.278
I–II	24	27		
**ER Status**				

Positive	20	22	0.727	0.573
Negative	10	8		
**PR Status**				

Positive	17	19	0.757	0.598
Negative	13	11		
**Her2 Status**				

Positive	16	7	3.755	0.017∗
Negative	14	23		
**Ki67**				

>14%	25	24	1.250	0.739
≤14%	5	6		

OR, odds ratio.

∗ p < 0.05.

**Figure 4:**
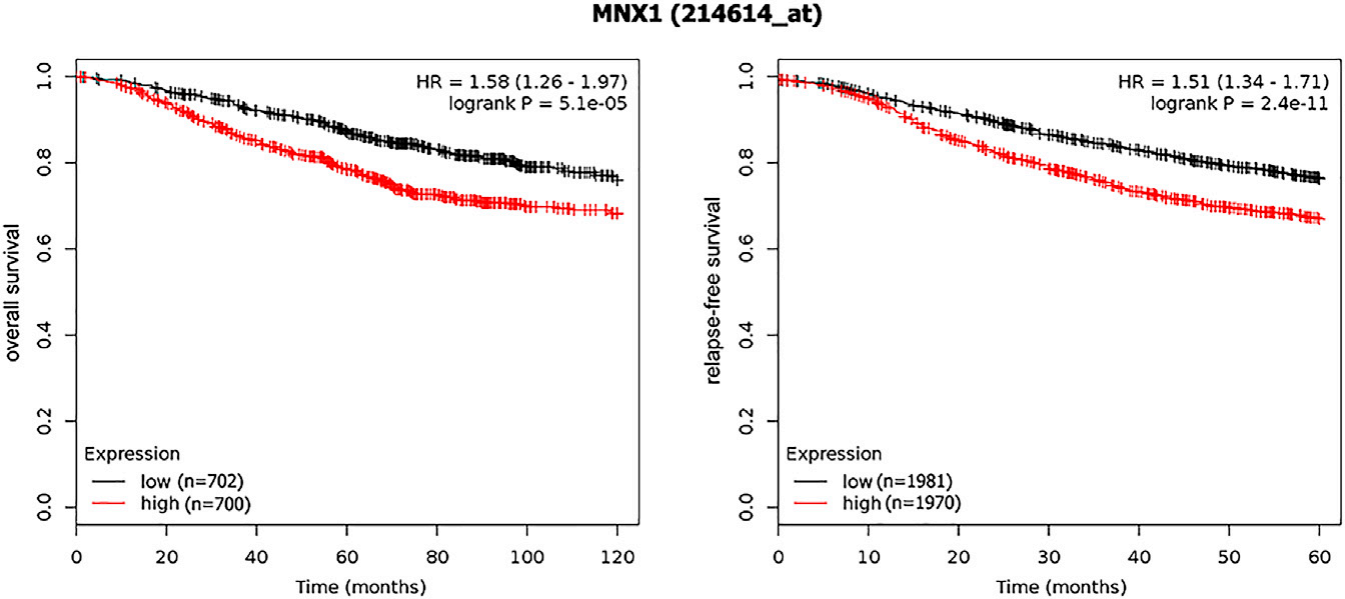
The Association between *MNX1* Expression and BC Survival Patients with higher *MNX1* expression had a poor survival compared to patients with lower *MNX1* expression.

As for survival, patients with higher *MNX1* expression showed a poorer overall survival (OS) (hazard ratio [HR] = 1.58; 95% confidence interval [CI], 1.26–1.97), as well as a worse relapse-free survival (RFS) (HR = 1.51; 95% CI, 1.34–1.71; Figure 4).

### *MNX1*-Correlated Genes and PPI Network

**Figure 5:**
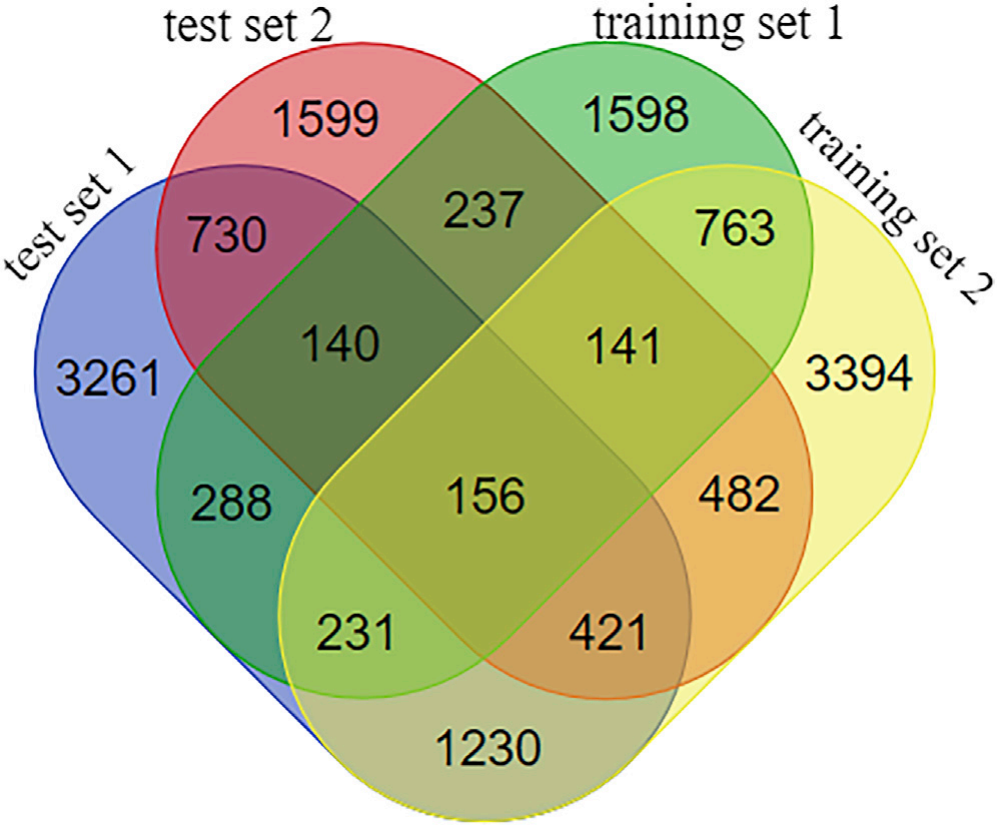
Venn Diagram of Overlapping Genes in the Four Gene Sets The overlapped genes were identified as significant *MNX1*-related DEGs in BC.

**Figure 6:**
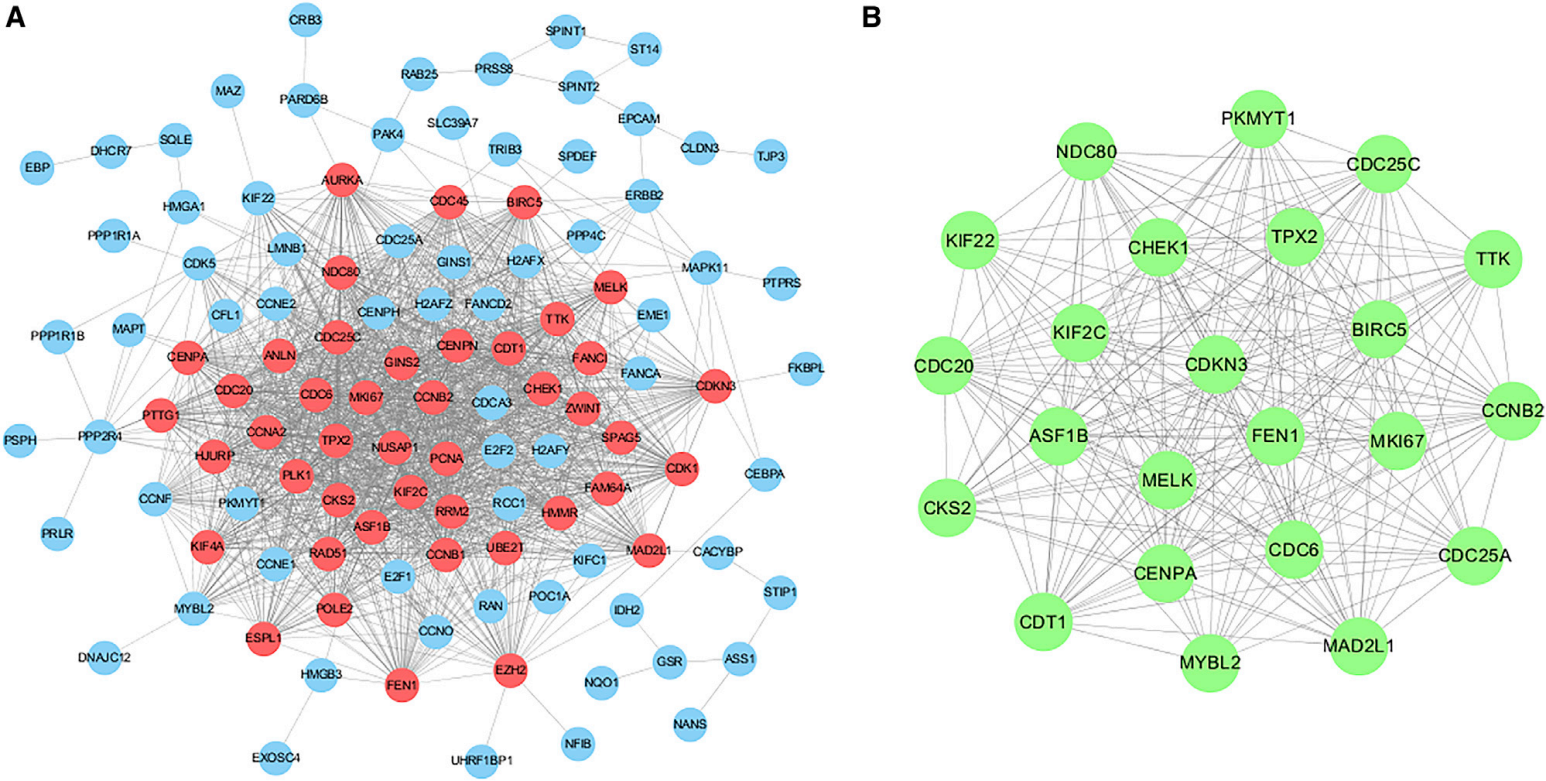
PPI Network of *MNX1*-Correlated Genes (A) Whole network of the interacting proteins in BC. The pink nodes denote the most highly interconnected proteins in the network. (B) Sub-network of hub genes. Each protein was interacted with more than 10 other proteins in the network.

A total of 156 significant *MNX1*-correlated genes in BC were identified according to our criteria (Figure 5; the four gene sets are presented in Table S1). The protein-protein interaction (PPI) network involved 113 nodes and 1,486 edges. As shown in Figure 6A, a majority of the nodes connected with three or more other nodes, suggesting that these proteins interacted closely. One module was discovered, comprising the 44 most highly interconnected nodes in the network (indicated in pink). Furthermore, 23 hub genes were identified, each of which interacted with more than 10 other proteins in the network. These hub genes were presented in a sub-network in Figure 6B.

**Figure 7:**
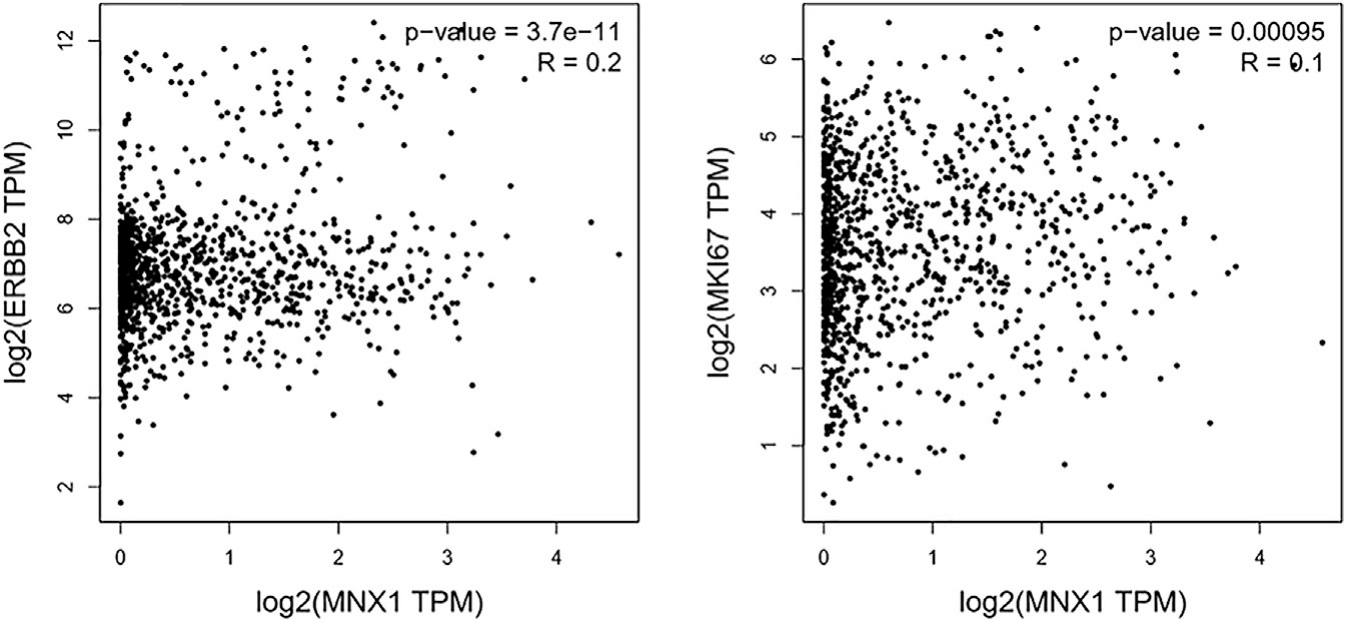
The Correlation of *MNX1* Expression with *ERBB2* (*HER2*) and *MKI67* Expression Pearson correlation coefficients were calculated using data from TCGA.

*MKI67*, one of the hub genes, is also an important biomarker for proliferation in BC. We specifically examined the correlation of *MNX1* with *MKI67* and *ERBB2* (also known as *HER2*) by Pearson correlation analysis using TCGA data. Consistently, both genes were significantly correlated with *MNX1* (Figures 7A and 7B).

### GO Function and Pathway Enrichment

**Figure 8:**
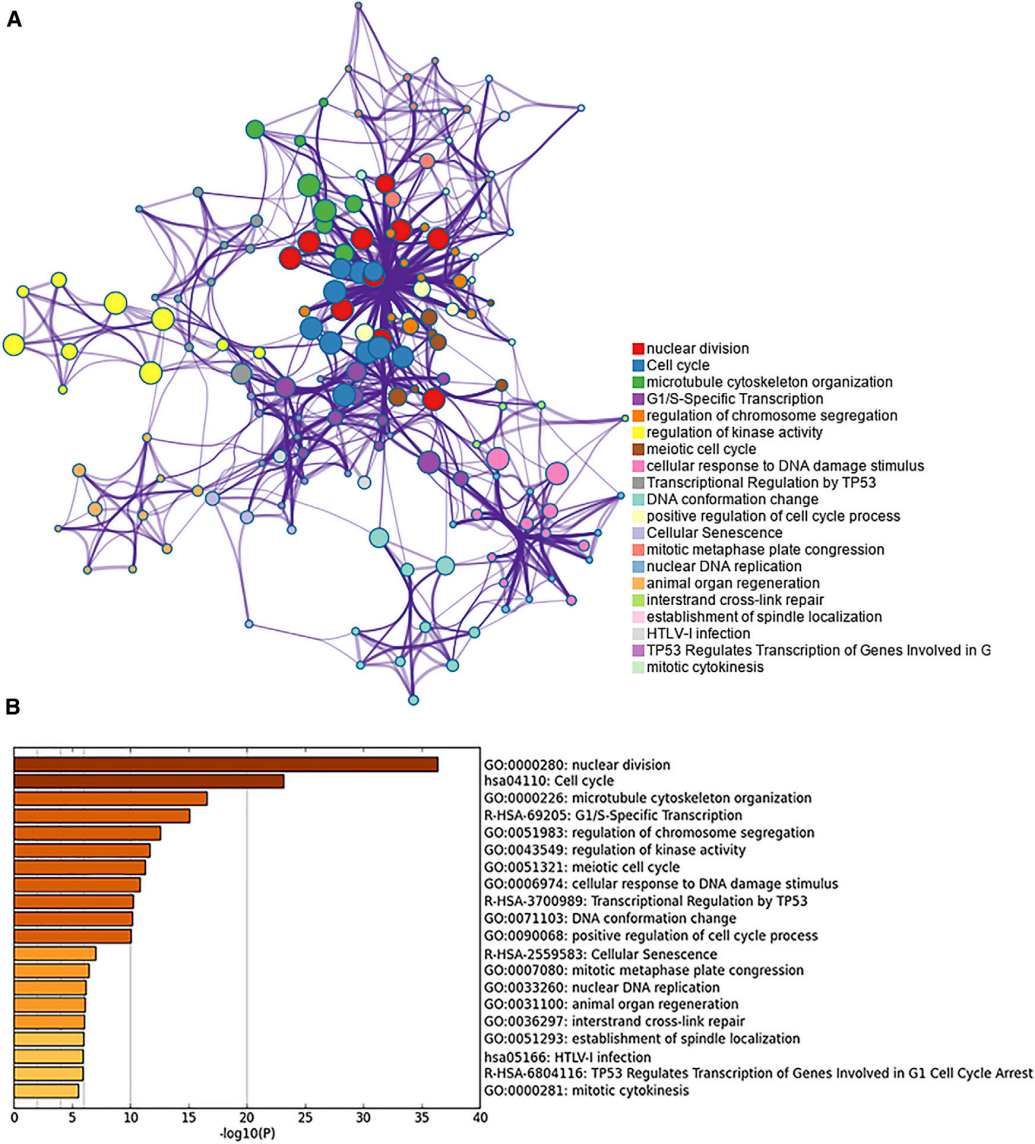
The 20 Top-Score Clusters of Significantly Enriched Terms (A) Network of the 20 clusters comprising the 10 best terms within each. Each node represents one enriched term, and each color represents a cluster. Terms paired with kappa similarity above 0.3 were connected. The thicker the edge displayed, the higher the similarity is. (B) Heatmap of the 20 top-score clusters. Each bar represents a cluster. The darker the color of the bar is, the smaller the p value is.

For an in-depth knowledge of the differentially expressed genes (DEGs), we conducted a functional enrichment analysis. The results indicated that the DEGs were significantly enriched in Gene Ontology (GO) terms, including nuclear division, chromosome segregation, and regulation of cell cycle, microtubule cytoskeleton organization, and regulation of kinase activity. These DEGs were also enriched in several cancer-related pathways such as the cell cycle, which was the most significantly enriched one, as well as the p53 signaling pathway, signaling by Rho GTPases, and pathways in cancer. The top 20 clusters of significantly enriched terms are shown in Figure 8. All the enriched pathways are summarized in Table S2.

## Discussion

BC is an extremely heterogeneous disease whose pathogenesis is rather complicated. Genetic factors play an important role in tumorigenesis and cancer progression.^15^ Next-generation sequencing has accelerated the implementation of genomic profiling in cancer patients. By far, a number of oncogenes and cancer suppressor genes have been discovered, some of which have been implicated in BC, including *TP53*, *HER2*, and *PIK3CA*.^16^, ^17^ However, there are still numerous unknown genes that may act as potential biomarkers for diagnosis or targets for treatment. Here, we identified a novel gene, *MNX1*, whose role in BC is unclear. Also, we made a primary exploration on its expression, clinical significance, and potential molecular function.

Our analysis demonstrated that *MNX1* was upregulated in BC tissues and correlated with several clinicopathological features of BC. The expression of *MNX1* was elevated in patients with larger tumor size and more lymph node metastasis. In addition, patients with higher *MNX1* expression had a poorer survival. These results indicate that *MNX1* may act as a cancer promoter in BC. Moreover, we found that *MNX1* expression correlated with Her2 status. The *MNX1* level was significantly higher in Her2-positive BC. This indicates that it may be a potential therapeutic target for this subtype of BC.

Previous studies have also shown that *MNX1* plays a role in several cancers. Zhang et al.^9^ reported *MNX1* to be an oncogene that was increased in prostate cancer, and its expression was regulated by the androgen and AKT signaling pathways. It can promote oncogenesis by stimulating the expression of downstream target genes *SREBP1* and *FASN*.^9^ In a microarray analysis of poorly differentiated HCC cell lines, *MNX1* was identified as the most upregulated gene. The upregulation was further confirmed by qRT-PCR in HCC tissues.^10^ Nagel et al.^18^ found that, in Hodgkin lymphoma, the phosphatidylinositol 3-kinase (PI3K) signaling pathway can promote the expression of *MNX1*, probably via E2F3. Then *MNX1* can drive IL6 expression and, thus, enhance the biological function of IL6.^18^ Desai et al.^13^, ^19^ found that GSK-3 stabilizes and phosphorylates MNX1 protein in insulinoma cells and that phospho-MNX1 can activate the oncogenic c-Met pathway by suppressing the c-Met inhibitor Cblb. All these lines of evidence suggested that *MNX1* is oncogenic in multiple cancer types. However, another study reported that *MNX1* has a dual role in childhood leukemia, as an oncogene in infant AML and as a tumor suppressor in childhood acute lymphoblastic leukemia (ALL).^20^ *MNX1* has been implicated in the development of both solid and hematological malignancies, although more investigations of the underlying mechanisms are needed to fully understand the role of *MNX1* in cancer biology.

The correlation analysis showed that *MNX1* was positively correlated with *MKI67*, which is a biomarker of proliferation. The functional enrichment analysis found that *MNX1* is involved in diverse biological processes, among which nuclear division is the most prominent. It also participates in the cell cycle and p53 signaling pathways. These findings implied that *MNX1* may have an impact on cell proliferation and, thus, also in tumor development. Most importantly, the expression of *MNX1* was significantly correlated with *HER2* expression. As *HER2* is a well-known crucial oncogene in BC, which activates multiple signaling pathways, including the mitogen-activated protein kinase (MAPK), PI3K/Akt, and STAT pathways, *MNX1* may serve as one of the target genes in these pathways.

In summary, our study suggests that *MNX1* may act as a tumor promoter in BC and that it may be involved in BC development through *HER2*-associated pathways or by regulating the cell cycle. The limitation of our study is that this study is just a primary exploration on the expression and clinical significance of *MNX1* in BC. We only made a functional prediction through bioinformatic analysis. Hence, further experiments on its function and mechanism are needed to clarify its specific role in BC, which is also the work for our next step. Nevertheless, we still hope that the findings of the present study will draw more attention to *MNX1* in BC research and shed a light on the direction of future studies.

## Materials and Methods

### Analysis of Public Data

The expression matrix of *MNX1* in breast tissues was obtained from the gene expression RNASeq (Illumina HiSeq) dataset of the BRCA cohort in the TCGA database. The raw data of gene expression level were log_2_(x + 1) transformed and processed at the University of California, Santa Cruz (UCSC) Xena repository. Reprocessed data were downloaded using the UCSC Xena Functional Genomics Explorer (https://xenabrowser.net/). The relationships between *MNX1* expression and clinical features of BC were also analyzed using TCGA data through the online platform LinkedOmics (http://www.linkedomics.org/).^21^

The impact of *MNX1* expression on BC survival was assessed through the online Kaplan-Meier Plotter tool (http://kmplot.com/analysis/).^22^ OS and RFS curves were made and compared by the log-rank test. HR and 95% CI were calculated to evaluate the correlation between *MNX1* level and BC survival.

### Patients and Tissue Samples

First, 33 pairs of BC tissue and normal breast tissue were obtained from the Second Affiliated Hospital of Xi’an Jiaotong University (Xi’an, China). Next, BC tissues of 27 other patients were collected. Specimens were put into RNAlater solution, immediately after surgery, and stored at −80°C. In addition, 3-mm-thick tumor tissues from 10 patients were fixed in formalin and then embedded in paraffin. All the patients were diagnosed with primary non-metastatic BC by pathology and did not receive chemotherapy or radiotherapy before surgery. The clinicopathological features were collected from patients, and each patient gave informed consent. The study was approved by the Ethics Committee of Xi’an Jiaotong University.

### RNA Isolation and qRT-PCR

Total RNA was extracted from tissue samples using TRIzol reagent (Invitrogen, Waltham, MA, USA) and was reverse transcribed into cDNA using the PrimeScript RT Reagent Kit (TaKaRa, Tokyo, Japan) following the manufacturer’s protocol. The expression levels of *MNX1* were determined by qRT-PCR using the SYBR Premix Ex Taq II Kit (TaKaRa, Tokyo, Japan) on the ABI StepOne Real-Time PCR system. GAPDH was used as an internal control. The primers are as follows: *MNX1* forward: 5′-CACTCGCGTGGGAGTTTGTG-3′; reverse: 5′-CCAATAATCAAAGTCGCCGCC-3′; GAPDH forward: 5′-GACAG TCAGCCGCATCTTCT-3′; reverse: 5′-GCGCCCAATACGACCAAATC-3′. The relative mRNA expression was calculated using the 2^−ΔΔCt^ method.

### Immunohistochemistry

Tissue sections were deparaffinized with xylene and rehydrated with graded alcohols. After antigen retrieval, 3% hydrogen peroxide was used to block the endogenous peroxidase activity. Then, slices were blocked by 10% goat plasma and incubated with 0.1% Triton X-100. Subsequently, the sections were incubated with anti-MNX1 antibody (5 μg/mL, Abcam, Cambridge, MA, USA) at 4°C overnight. After incubation with biotinylated secondary antibody and horseradish-peroxidase-labeled streptavidin, the sections were detected by DAB and counterstained with hematoxylin. Images were captured on the Nikon Eclipse 50i microscope.

### Statistical Analysis

Statistical tests were carried out using IBM SPSS Statistics 22.0 software. The difference in *MNX1* expression between BC tissues and normal breast tissues was examined by Welch’s t test. A chi-square test was applied to evaluate the association of *MNX1* expression with BC clinicopathological features.

### Mining Correlated Genes

Prospective correlated genes of *MNX1* were analyzed through LinkedOmics using the Pearson correlation test. Genes with a p value < 0.01 and correlation coefficient (corrcoef) ≥ 0.1 were chosen to form the test set. In order to increase the credibility, we used the *MNX1*-related genes mined from the Multi Experiment Matrix (MEM) database (https://biit.cs.ut.ee/mem/) as another test set.^23^ In addition, we mined DEGs in BC through GEPIA (http://gepia.cancer-pku.cn/).^24^ Fold change (FC) ≥ 1 and p value < 0.01 were defined as the cutoff to select significant DEGs, which consist of a training set. We also used PALM-IST (http://www.hpppi.iicb.res.in/ctm/index.html) to mine BC-associated genes from the literature, which also formed a training set. The overlapping genes of the four sets were finally identified as significant *MNX1*-related DEGs in BC. The Venn diagram for overlapping genes was drawn by an online tool (http://bioinformatics.psb.ugent.be/webtools/Venn/).

### Protein Interaction Analysis

The PPIs for the *MNX1* and its correlated genes were analyzed by STRING (v.10.5; http://www.string-db.org/).^25^ A combined score of ≥0.4 was used as the cutoff for significant interaction. The PPI network was then visualized using the software Cytoscape (v.3.5.1). Module analysis was carried out by MCODE, with the criteria of a degree ≥2 and k-core ≥3. Hub genes were selected by cytoHubba.^26^ As there are 12 topological algorithms, we used the median as the cutoff to find the overlapping genes according to their score under each algorithm, which were designated as hub genes.

### Functional Enrichment Analysis

Functional enrichment analysis for *MNX1*-correlated genes along with *MNX1* was performed by Metascape (http://metascape.org).^27^ The enrichment analysis comprises the GO biological process (BP), Kyoto Encyclopedia of Genes and Genomes (KEGG) pathways, and Reactome pathways. Only terms with p values < 0.01 and a number of enriched genes ≥3 were considered as significant. All the resultant terms were then grouped into clusters, based on their similarities. The most enriched term within a cluster was chosen as the one to represent the cluster.

## Author Contributions

T.T. and Z.-J.D. conceived and designed the study. T.T., M.W., and Y.Z. retrieved databases and collected data. S.L., C.D., and Y.D. organized and collated data. T.T. and T.Y. analyzed and interpreted data. T.T. and D.S. performed experiments. N.L. and Z.Z. prepared tables and figures. T.T. and M.W. drafted the manuscript. Z.-J.D., W.Z., and H.L. revised the manuscript. All authors approved the final manuscript.

## Conflicts of Interest

The authors have no conflicts of interest.

## Supplemental Information

Document S2. Tables S1

Document S3. Article plus Supplemental Information
