# Supplementary material for: Expression, Clinical Significance, and Functional Prediction of MNX1 in Breast Cancer
Source: Mol Ther Nucleic Acids. 2018 Sep 27;13:399–406. doi: 10.1016/j.omtn.2018.09.014 (PMC6205149; doi:10.1016/j.omtn.2018.09.014)
Supplement: Document S1. Tables S2 [file mmc1.pdf]

OMTN, Volume *13*

Supplemental Information

Expression, Clinical Significance, and Functional Prediction of *MNX1* in Breast Cancer

Tian Tian, Meng Wang, Yuyao Zhu, Wenge Zhu, Tielin Yang, Hongtao Li, Shuai Lin, Cong Dai, Yujiao Deng, Dingli Song, Na Li, Zhen Zhai, and Zhi-Jun Dai

Supplementary Table2. The enriched pathways

Category	Term	P-value	Count
Reactome	R-HSA-1640170: Cell Cycle	1.00E-31	43
Reactome	R-HSA-69278: Cell Cycle, Mitotic	1.00E-30	40
KEGG	hsa04110: Cell cycle	1.00E-23	21
Reactome	R-HSA-69205: G1/S-Specific Transcription	1.00E-15	9
Reactome	R-HSA-113510: E2F mediated regulation of DNA replication	1.00E-14	10
Reactome	R-HSA-1538133:G0 and Early G1	1.00E-13	9
Reactome	R-HSA-453279: Mitotic G1-G1/S phases	1.00E-13	15
Reactome	R-HSA-69273: Cyclin A/B1 associated events during G2/M transition	1.00E-12	8
Reactome	R-HSA-2500257: Resolution of Sister Chromatid Cohesion	1.00E-12	13
Reactome	R-HSA-69206: G1/S Transition	1.00E-12	13
KEGG	hsa04114: Oocyte meiosis	1.00E-12	13
Reactome	R-HSA-68877: Mitotic Prometaphase	1.00E-12	13
Reactome	R-HSA-68886: M Phase	1.00E-11	18
Reactome	R-HSA-69620: Cell Cycle Checkpoints	1.00E-11	15
Reactome	R-HSA-156711: Polo-like kinase mediated events	1.00E-11	7
Reactome	R-HSA-6791312: TP53 Regulates Transcription of Cell Cycle Genes	1.00E-11	9
Reactome	R-HSA-3700989: Transcriptional Regulation by TP53	1.00E-10	18
Reactome	R-HSA-453274: Mitotic G2-G2/M phases	1.00E-10	14
Reactome	R-HSA-69242: S Phase	1.00E-10	12
Reactome	R-HSA-69306: DNA Replication	2.00E-10	11
Reactome	R-HSA-69275: G2/M Transition	6.31E-10	13
Reactome	R-HSA-68882: Mitotic Anaphase	7.94E-10	13
Reactome	R-HSA-2555396: Mitotic Metaphase and Anaphase	7.94E-10	13
KEGG	hsa04914: Progesterone-mediated oocyte maturation	7.94E-10	10
Reactome	R-HSA-2467813: Separation of Sister Chromatids	5.01E-09	12
Reactome	R-HSA-69478: G2/M DNA replication checkpoint	1E-08	4
Reactome	R-HSA-157881: Cyclin B2 mediated events	1E-08	4
Reactome	R-HSA-5663220: RHO GTPases Activate Formins	2.00E-08	10
Reactome	R-HSA-195258: RHO GTPase Effectors	2.00E-08	14
Reactome	R-HSA-176417: Phosphorylation of Emi1	2.51E-08	4
Reactome	R-HSA-69239: Synthesis of DNA	3.16E-08	9
Reactome	R-HSA-73886: Chromosome Maintenance	5.01E-08	9
Reactome	R-HSA-2980767: Activation of NIMA Kinases NEK9, NEK6, NEK7	6.31E-08	4
Reactome	R-HSA-2559583: Cellular Senescence	1E-07	11
Reactome	R-HSA-6804114: TP53 Regulates Transcription of Genes Involved in G2 Cell Cycle Arrest	1.26E-07	5
Reactome	R-HSA-453276: Regulation of mitotic cell cycle	1.26E-07	8
Reactome	R-HSA-174143: APC/C-mediated degradation of cell cycle proteins	1.26E-07	8
Reactome	R-HSA-73894: DNA Repair	2.00E-07	13

Reactome	R-HSA-69481G: 2/M Checkpoints	2.51E-07	10
KEGG	**hsa04115: p53 signaling pathway**	3.98E-07	7
Reactome	R-HSA-5693538: Homology Directed Repair	3.98E-07	9
Reactome	R-HSA-75035: Chk1/Chk2(Cds1) mediated inactivation of Cyclin B:Cdk1 complex	7.94E-07	4
Reactome	R-HSA-176814: Activation of APC/C and APC/C:Cdc20 mediated degradation of mitotic proteins	0.000001	7
Reactome	R-HSA-2559586: DNA Damage/Telomere Stress Induced Senescence	0.000001	7
KEGG	hsa05166: HTLV-I infection	1.26E-06	11
Reactome	R-HSA-6804116: TP53 Regulates Transcription of Genes Involved in G1 Cell Cycle Arrest	1.26E-06	4
Reactome	**R-HSA-194315: Signaling by Rho GTPases**	1.58E-06	14
KEGG	hsa03460: Fanconi anemia pathway	1.58E-06	6
Reactome	R-HSA-162658: Golgi Cisternae Pericentriolar Stack Reorganization	1.58E-06	4
Reactome	R-HSA-5693532: DNA Double-Strand Break Repair	2.00E-06	9
Reactome	R-HSA-69190: DNA strand elongation	2.00E-06	5
Reactome	R-HSA-69298: Association of licensing factors with the pre-replicative complex	2.51E-06	4
Reactome	R-HSA-5693567: HDR through Homologous Recombination (HR) or Single Strand Annealing (SSA)	3.16E-06	8
Reactome	R-HSA-176187: Activation of ATR in response to replication stress	3.98E-06	5
KEGG	**hsa05206: MicroRNAs in cancer**	5.01E-06	11
Reactome	R-HSA-6783310: Fanconi Anemia Pathway	6.31E-06	5
Reactome	R-HSA-606279: Deposition of new CENPA-containing nucleosomes at the centromere	0.00001	6
Reactome	R-HSA-774815: Nucleosome assembly	0.00001	6
Reactome	R-HSA-176409: APC/C:Cdc20 mediated degradation of mitotic proteins	1.26E-05	6
Reactome	R-HSA-8863678: Neurodegenerative Diseases	1.58E-05	4
Reactome	R-HSA-8862803: Deregulated CDK5 triggers multiple neurodegenerative pathways in Alzheimer's disease models	1.58E-05	4
Reactome	R-HSA-176408: Regulation of APC/C activators between G1/S and early anaphase	2.00E-05	6
Reactome	R-HSA-2980766: Nuclear Envelope Breakdown	2.00E-05	5
Reactome	R-HSA-68874: M/G1 Transition	2.51E-05	6
Reactome	R-HSA-69002: DNA Replication Pre-Initiation	2.51E-05	6
Reactome	R-HSA-113507: E2F-enabled inhibition of pre-replication complex	3.16E-05	3
Reactome	**R-HSA-6804756: Regulation of TP53 Activity through Phosphorylation**	3.98E-05	6
Reactome	R-HSA-68875: Mitotic Prophase	3.98E-05	7
Reactome	R-HSA-68689: CDC6 association with the ORC:origin complex	5.01E-05	3

Reactome	R-HSA-2514853: Condensation of Prometaphase Chromosomes	5.01E-05	3
KEGG	hsa05161: Hepatitis B	5.01E-05	7
Reactome	R-HSA-2262752: Cellular responses to stress	5.01E-05	12
Reactome	R-HSA-68962: Activation of the pre-replicative complex	6.31E-05	4
Reactome	R-HSA-176974: Unwinding of DNA	6.31E-05	3
Reactome	R-HSA-69615: G1/S DNA Damage Checkpoints	0.0001	5
Reactome	R-HSA-5685942: HDR through Homologous Recombination (HRR)	0.0001	5
Reactome	**R-HSA-5633007: Regulation of TP53 Activity**	0.0001	7
Reactome	R-HSA-4419969: Depolymerisation of the Nuclear Lamina	0.000126	3
Reactome	R-HSA-8854518: AURKA Activation by TPX2	0.000126	5
Reactome	R-HSA-2299718: Condensation of Prophase Chromosomes	0.000126	5
Reactome	R-HSA-69304: Regulation of DNA replication	0.0002	5
Reactome	R-HSA-8852276: The role of GTSE1 in G2/M progression after G2 checkpoint	0.0002	5
Reactome	R-HSA-2559580: Oxidative Stress Induced Senescence	0.0002	6
Reactome	R-HSA-157579: Telomere Maintenance	0.000251	5
KEGG	**hsa05222: Small cell lung cancer**	0.000251	5
Reactome	R-HSA-176412: Phosphorylation of the APC/C	0.000251	3
KEGG	**hsa05215: Prostate cancer**	0.000316	5
KEGG	hsa00100: Steroid biosynthesis	0.000316	3
Reactome	R-HSA-2565942: Regulation of PLK1 Activity at G2/M Transition	0.000316	5
Reactome	R-HSA-5651801: PCNA-Dependent Long Patch Base Excision Repair	0.000316	3
KEGG	hsa01522: Endocrine resistance	0.000398	5
Reactome	R-HSA-69473: G2/M DNA damage checkpoint	0.000398	5
KEGG	hsa00480: Glutathione metabolism	0.000501	4
Reactome	R-HSA-174048: APC/C:Cdc20 mediated degradation of Cyclin B	0.000501	3
Reactome	R-HSA-110373: Resolution of AP sites via the multiple-nucleotide	0.000501	3
Reactome	R-HSA-191273: Cholesterol biosynthesis	0.000501	3
Reactome	R-HSA-174417: Telomere C-strand (Lagging Strand) Synthesis	0.000501	3
Reactome	R-HSA-983189: Kinesins	0.000631	4
KEGG	**hsa05212: Pancreatic cancer**	0.000794	4
KEGG	hsa04530: Tight junction	0.001	6
Reactome	R-HSA-180786: Extension of Telomeres	0.001	3
Reactome	R-HSA-420029: Tight junction interactions	0.001	3
Reactome	R-HSA-68867: Assembly of the pre-replicative complex	0.001259	4
KEGG	**hsa05200: Pathways in cancer**	0.001259	9
KEGG	hsa03410: Base excision repair	0.001259	3
Reactome	R-HSA-69656: Cyclin A:Cdk2-associated events at S phase entry	0.001259	4
Reactome	R-HSA-3301854: Nuclear Pore Complex (NPC) Disassembly	0.001585	3
Reactome	R-HSA-6920: Cyclin E associated events during G1/S transition	0.001585	4
Reactome	R-HSA-8856688: Golgi-to-ER retrograde transport	0.001585	5

Reactome	R-HSA-174184: Cdc20:Phospho-APC/C mediated degradation of Cyclin A	0.001585	4
Reactome	R-HSA-174178: APC/C:Cdh1 mediated degradation of Cdc20 and other APC/C:Cdh1 targeted proteins in late mitosis/earl	0.001585	4
Reactome	R-HSA-179419: APC:Cdc20 mediated degradation of cell cycle proteins prior to satisfation of the cell cycle checkpo	0.001585	4
KEGG	hsa03030: DNA replication	0.001585	3
Reactome	R-HSA-73884: Base Excision Repair	0.001995	3
Reactome	R-HSA-73933: Resolution of Abasic Sites (AP sites)	0.001995	3
KEGG	**hsa05203: Viral carcinogenesis**	0.001995	6
KEGG	**hsa05219: Bladder cancer**	0.002512	3
Reactome	R-HSA-4615885: SUMOylation of DNA replication proteins	0.003162	3
Reactome	R-HSA-6811434: COPI-dependent Golgi-to-ER retrograde traffic	0.003981	4
Reactome	R-HSA-5693607: Processing of DNA double-strand break ends	0.003981	4
KEGG	**hsa05223: Non-small cell lung cancer**	0.005012	3
Reactome	R-HSA-983231: Factors involved in megakaryocyte development and platelet production	0.00631	5
Reactome	R-HSA-1655829: Regulation of cholesterol biosynthesis by SREBP (SREBF)	0.00631	3
KEGG	hsa04360: Axon guidance	0.00631	5
Reactome	R-HSA-187577: SCF(Skp2)-mediated degradation of p27/p21	0.007943	3
Reactome	R-HSA-421270: Cell-cell junction organization	0.007943	3
Reactome	R-HSA-1500620: Meiosis	0.007943	4
Reactome	R-HSA-5334118: DNA methylation	0.01	3
Reactome	R-HSA-6811442: Intra-Golgi and retrograde Golgi-to-ER traffic	0.01	5
Reactome	R-HSA-69580: p53-Dependent G1/S DNA damage checkpoint	0.01	3
Reactome	R-HSA-69563: p53-Dependent G1 DNA Damage Response	0.01	3
