# Supplementary material for: Expression, Clinical Significance, and Functional Prediction of MNX1 in Breast Cancer
Source: Mol Ther Nucleic Acids. 2018 Sep 27;13:399–406. doi: 10.1016/j.omtn.2018.09.014 (PMC6205149; doi:10.1016/j.omtn.2018.09.014)
Supplement: Document S3. Article plus Supplemental Information [file mmc3.pdf]

Molecular Therapy
Nucleic Acids
Original Article

AMERICAN SOCIETY of GENE & CELL THERAPY

Expression, Clinical Significance, and Functional Prediction of *MNX1* in Breast Cancer

Tian Tian,[1,2,6] Meng Wang,[1,2,6] Yuyao Zhu,[1] Wenge Zhu,[3] Tielin Yang,[4] Hongtao Li,[5] Shuai Lin,[1] Cong Dai,[1] Yujiao Deng,[1] Dingli Song,[1] Na Li,[1] Zhen Zhai,[1] and Zhi-Jun Dai[1,2]

[1]Department of Breast Surgery, Guangzhou Women and Children's Medical Center, Guangzhou Medical University, Guangzhou 510623, Guangdong, China; [2]Department of Oncology, The Second Affiliated Hospital of Xi'an Jiaotong University, Xi'an 710004, China; [3]Department of Biochemistry and Molecular Medicine, The George Washington University Medical School, Washington, DC 20052, USA; [4]School of Life Science and Technology, Xi'an Jiaotong University, Xi'an 710049, China; [5]Department of Breast, Head and Neck Surgery, Affiliated Tumor Hospital of Xinjiang Medical University, Urumchi 830000, China

Motor neuron and pancreas homeobox 1 (*MNX1*) is a key developmental gene. Previous studies found that it was upregulated in several tumors, but its role in breast cancer (BC) remains unclear. In order to have a better understanding of this gene in BC, we examined the expression of *MNX1* in BC tissues and normal breast tissues by qRT-PCR and by analyzing data from The Cancer Genome Atlas (TCGA) database. We also assessed the association of *MNX1* expression with BC clinicopathological features and investigated the impact of *MNX1* on BC survival. Potential molecular function of *MNX1* was predicted through protein-protein interactions and functional enrichment. The results showed that the expression of *MNX1* was significantly increased in BC tissues, especially in the HER2-positive subtype, and *MNX1* expression was associated with several clinical characteristics, including menopause status, receptor status, subtypes, tumor size, lymph node metastasis, and race. In addition, patients with higher *MNX1* expression had poorer survival. Enrichment analysis suggested that *MNX1* is probably involved in biological processes and pathways related to nuclear division, cell cycle, and p53 signaling. In conclusion, our study suggests that *MNX1* may act as a tumor promoter in BC. We hope these findings will draw more attention to *MNX1* in future cancer studies.

INTRODUCTION

Breast cancer (BC) is the most common cancer type and the leading cause of global cancer death among females.[1] Although cancer treatment has reached the era of precision medicine, the precise treatment of BC remains a challenge, because no effective targets have been found except for a few validated biomarkers, such as estrogen receptor (ER), HER2, PIK3CA, and AKT1. Currently, the application of targeted therapy in BC is limited.[2] Therefore, the study of molecular mechanisms underlying BC and novel oncogenic drivers is important, which may lead to the identification of potential therapeutic targets for BC.

MNX1, located on human chromosome 7q36.3, belongs to the family of homeobox genes. It encodes a nuclear protein named motor neuron and pancreas homeobox 1 (MNX1), also known as HLXB9 or HB9, which is a transcription factor.[3] *MNX1* is a key developmental gene that is normally expressed in neurons as well as pancreatic and lymphoid cells. It is involved in both motor neuronal differentiation and pancreatic beta cell development.[4–6] Defects in this gene result in hereditary sacral agenesis, which is also called Currarino syndrome.[7,8] The function of *MNX1* in cancer biology has not been clarified. However, the expression of *MNX1* has been reported to be upregulated in several tumors, including prostate cancer, hepatocellular carcinoma (HCC), acute myeloid leukemia (AML), and neuroblastoma.[3,9–11] Furthermore, it has been demonstrated to be oncogenic in prostate cancer and insulinoma.[12,13]

Although evidence has shown that *MNX1* may play a role in tumorigenesis, its expression and function in BC is still unclear by far. This gene had hardly been studied in BC before. Only Neufing et al.[14] have reported that the percentage of nuclei expressing of MNX1 is increased in breast carcinoma. However, the intensity of nuclear staining is decreased progressively with increasing tumor grade. In order to have a better understanding of *MNX1*in BC, we investigated its expression profile and clinical significance in BC and the impact of its expression on BC survival while also exploring the potential molecular function through bioinformatic analysis and experimental method.

RESULTS

MNX1 Expression Is Upregulated in BC Tissues

We explored the expression profile of *MNX1* in BC tissues using The Cancer Genome Atlas (TCGA) dataset. There were 1,218 samples in the dataset, including 1,104 BC tissues and 114 normal breast tissues. The results indicated that, compared with the normal breast

Received 29 December 2017; accepted 23 September 2018;
https://doi.org/10.1016/j.omtn.2018.09.014.

[6]These authors contributed equally to this work.

Correspondence: Zhi-Jun Dai, Department of Breast Surgery, Guangzhou Women and Children's Medical Center, Guangzhou Medical University, Guangzhou 510623, Guangdong, China.
E-mail: dzj0911@xjtu.edu.cn

This is an open access article under the CC BY license (http://creativecommons.org/licenses/by/4.0/).

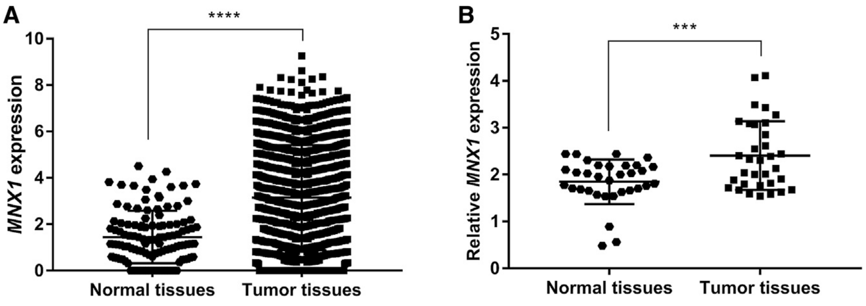

Figure 1. The Expression of *MNX1* Was Significantly Increased in BC Tissues compared with Normal Breast Tissues

(A) Analysis of *MNX1* expression profile using data from TCGA. (B) *MNX1* expression was detected by qRT-RCR in 33 pairs of BC tissues and normal breast tissues. ***p < 0.0001 and ****p = 0.0006.

tissues, the level of *MNX1* was significantly increased in BC tissues ($p < 0.0001$; Figure 1A). Similarly, qRT-PCR results also revealed that *MNX1* expression was significantly upregulated in BC tissues ($p = 0.0006$; Figure 1B). Immunohistochemistry showed that MNX1 protein was mainly expressed in the nucleus of BC cells (Figure 2).

MNX1 Expression Is Associated with BC Clinicopathology and Survival

The relationship between *MNX1* expression and BC clinicopathological features was assessed in 60 BC patients. The median *MNX1* expression of all BC tissues was used as the cutoff value to divide BC patients into two groups. As shown in Table 1, *MNX1* expression was related to menopause status and Her2 expression. We subsequently analyzed the data from TCGA. We observed that the level of *MNX1* was higher in Her2-positive and progesterone receptor (PR)-negative tissues (Figures 3A and 3B). Consistently, *MNX1* was significantly upregulated in Her2-positive BC compared with that in the other three subtypes of BC (Figure 3C). In addition, *MNX1* level differs with race. The expression in Asians was the highest (Figure 3D). Moreover, patients with advanced tumor (T) and lymph node (N) stages tend to have an increased *MNX1* level (Figures 3E and 3F).

As for survival, patients with higher *MNX1* expression showed a poorer overall survival (OS) (hazard ratio [HR] = 1.58; 95% confidence interval [CI], 1.26–1.97), as well as a worse relapse-free survival (RFS) (HR = 1.51; 95% CI, 1.34–1.71; Figure 4).

MNX1-Correlated Genes and PPI Network

A total of 156 significant *MNX1*-correlated genes in BC were identified according to our criteria (Figure 5; the four gene sets are presented in Table S1). The protein-protein interaction (PPI) network involved 113 nodes and 1,486 edges. As shown in Figure 6A, a majority of the nodes connected with three or more other nodes, suggesting that these proteins interacted closely. One module was discovered, comprising the 44 most highly interconnected nodes in the network (indicated in pink). Furthermore, 23 hub genes were identified, each of which interacted with more than 10 other proteins in the network. These hub genes were presented in a sub-network in Figure 6B.

MKI67, one of the hub genes, is also an important biomarker for proliferation in BC. We specifically examined the correlation of *MNX1* with *MKI67* and *ERBB2* (also known as *HER2*) by Pearson correlation analysis using TCGA data. Consistently, both genes were significantly correlated with *MNX1* (Figures 7A and 7B).

GO Function and Pathway Enrichment

For an in-depth knowledge of the differentially expressed genes (DEGs), we conducted a functional enrichment analysis. The results indicated that the DEGs were significantly enriched in Gene Ontology (GO) terms, including nuclear division, chromosome segregation, and regulation of cell cycle, microtubule cytoskeleton organization, and regulation of kinase activity. These DEGs were also enriched in several cancer-related pathways such as the cell cycle, which was the most significantly enriched one, as well as

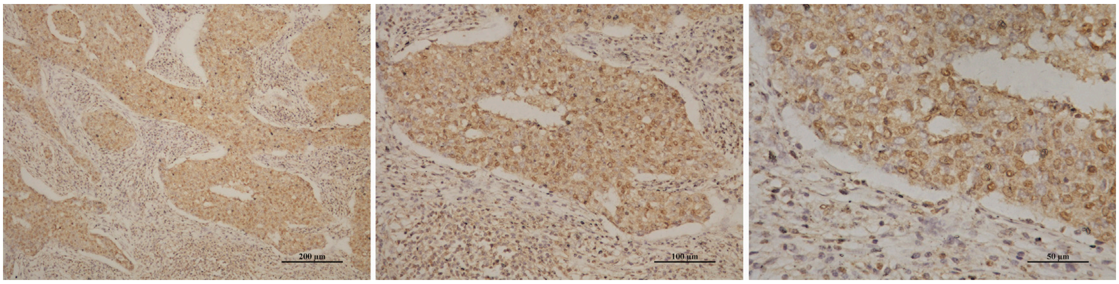

Figure 2. Representative Microscopy Images of BC Sections

Images are at (left) 10×, (middle) 20×, and (right) 40× magnifications, respectively, under the same field.

Table 1. Relationship between *MNX1* Expression and Clinicopathological Features of Breast Cancer Patients

Clinicopathological Parameters	*MNX1* Expression High (30)	*MNX1* Expression Low (30)	OR	p Value
Age (Years)				
55	14	10	1.750	0.292
≤55	16	20		
Menopause Status				
Postmenopausal	22	14	3.143	0.035*
Premenopausal	8	16		
Tumor Size				
>2 cm	18	13	1.962	0.196
≤2 cm	12	17		
Lymph Node Metastasis				
Positive	21	16	2.042	0.184
Negative	9	14		
TNM Stage				
III–IV	6	3	2.250	0.278
I–II	24	27		
ER Status				
Positive	20	22	0.727	0.573
Negative	10	8		
PR Status				
Positive	17	19	0.757	0.598
Negative	13	11		
Her2 Status				
Positive	16	7	3.755	0.017*
Negative	14	23		
Ki67				
>14%	25	24	1.250	0.739
≤14%	5	6		

OR, odds ratio.
*$p < 0.05$.

the p53 signaling pathway, signaling by Rho GTPases, and pathways in cancer. The top 20 clusters of significantly enriched terms are shown in Figure 8. All the enriched pathways are summarized in Table S2.

DISCUSSION

BC is an extremely heterogeneous disease whose pathogenesis is rather complicated. Genetic factors play an important role in tumorigenesis and cancer progression.[15] Next-generation sequencing has accelerated the implementation of genomic profiling in cancer patients. By far, a number of oncogenes and cancer suppressor genes have been discovered, some of which have been implicated in BC, including *TP53*, *HER2*, and *PIK3CA*.[16,17] However, there are still numerous unknown genes that may act as potential biomarkers for diagnosis or targets for treatment. Here, we identified a novel gene, *MNX1*, whose role in BC is unclear. Also, we made a primary exploration on its expression, clinical significance, and potential molecular function.

Our analysis demonstrated that *MNX1* was upregulated in BC tissues and correlated with several clinicopathological features of BC. The expression of *MNX1* was elevated in patients with larger tumor size and more lymph node metastasis. In addition, patients with higher *MNX1* expression had a poorer survival. These results indicate that *MNX1* may act as a cancer promoter in BC. Moreover, we found that *MNX1* expression correlated with Her2 status. The *MNX1* level was significantly higher in Her2-positive BC. This indicates that it may be a potential therapeutic target for this subtype of BC.

Previous studies have also shown that *MNX1* plays a role in several cancers. Zhang et al.[9] reported *MNX1* to be an oncogene that was increased in prostate cancer, and its expression was regulated by the androgen and AKT signaling pathways. It can promote oncogenesis by stimulating the expression of downstream target genes *SREBP1* and *FASN*.[9] In a microarray analysis of poorly differentiated HCC cell lines, *MNX1* was identified as the most upregulated gene. The upregulation was further confirmed by qRT-PCR in HCC tissues.[10] Nagel et al.[18] found that, in Hodgkin lymphoma, the phosphatidylinositol 3-kinase (PI3K) signaling pathway can promote the expression of *MNX1*, probably via E2F3. Then *MNX1* can drive IL6 expression and, thus, enhance the biological function of IL6.[18] Desai et al.[13,19] found that GSK-3 stabilizes and phosphorylates MNX1 protein in insulinoma cells and that phospho-MNX1 can activate the oncogenic c-Met pathway by suppressing the c-Met inhibitor Cblb. All these lines of evidence suggested that *MNX1* is oncogenic in multiple cancer types. However, another study reported that *MNX1* has a dual role in childhood leukemia, as an oncogene in infant AML and as a tumor suppressor in childhood acute lymphoblastic leukemia (ALL).[20] *MNX1* has been implicated in the development of both solid and hematological malignancies, although more investigations of the underlying mechanisms are needed to fully understand the role of *MNX1* in cancer biology.

The correlation analysis showed that *MNX1* was positively correlated with *MKI67*, which is a biomarker of proliferation. The functional enrichment analysis found that *MNX1* is involved in diverse biological processes, among which nuclear division is the most prominent. It also participates in the cell cycle and p53 signaling pathways. These findings implied that *MNX1* may have an impact on cell proliferation and, thus, also in tumor development. Most importantly, the expression of *MNX1* was significantly correlated with *HER2* expression. As *HER2* is a well-known crucial oncogene in BC, which activates multiple signaling pathways, including the mitogen-activated protein kinase (MAPK), PI3K/Akt, and STAT pathways, *MNX1* may serve as one of the target genes in these pathways.

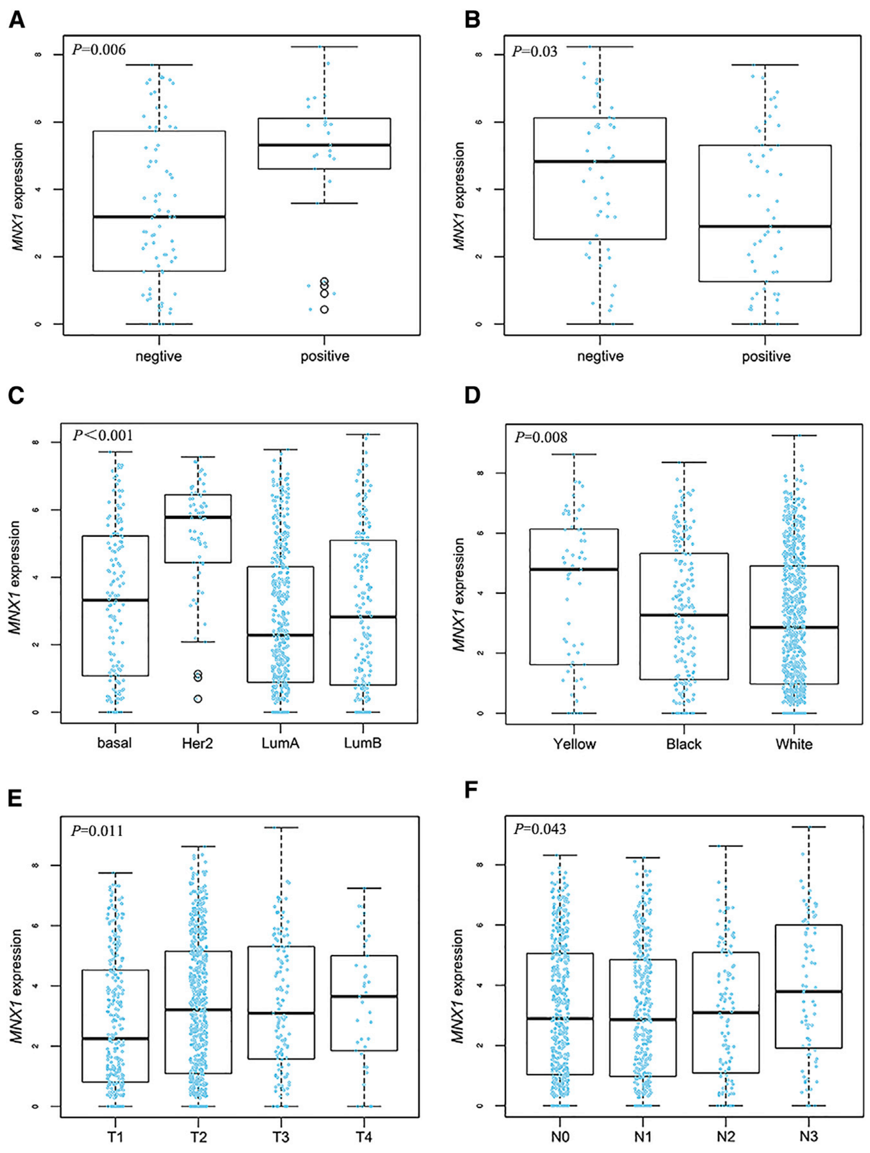

Figure 3. The Relationship between *MNX1* Expression and BC Clinical Features: Analyses of Data from TCGA

(A) *MNX1* expression and Her2 status. (B) *MNX1* expression and PR status. (C) *MNX1* expression in different subtypes. (D) *MNX1* expression in different races. (E) *MNX1* expression and T stage. (F) *MNX1* expression and N stage.

In summary, our study suggests that *MNX1* may act as a tumor promoter in BC and that it may be involved in BC development through *HER2*-associated pathways or by regulating the cell cycle. The limitation of our study is that this study is just a primary exploration on the expression and clinical significance of *MNX1* in BC. We only made a functional prediction through bioinformatic analysis. Hence, further experiments on its function and mechanism are needed to clarify its specific role in BC, which is also the work for our next step. Nevertheless, we still hope that the findings of the present study will draw more attention to *MNX1* in BC research and shed a light on the direction of future studies.

MATERIALS AND METHODS

Analysis of Public Data

The expression matrix of *MNX1* in breast tissues was obtained from the gene expression RNASeq (Illumina HiSeq) dataset of the BRCA cohort in the TCGA database. The raw data of gene expression level were $\log_2(x + 1)$ transformed and processed at the University of California, Santa Cruz (UCSC) Xena repository. Reprocessed data were downloaded using the UCSC Xena Functional Genomics Explorer (https://xenabrowser.net/). The relationships between *MNX1* expression and clinical features of BC were also analyzed using TCGA data through the online platform LinkedOmics (http://www.linkedomics.org/).[21]

The impact of *MNX1* expression on BC survival was assessed through the online Kaplan-Meier Plotter tool (http://kmplot.com/analysis/).[22] OS and RFS curves were made and compared by the log-rank test. HR and 95% CI were calculated to evaluate the correlation between *MNX1* level and BC survival.

Patients and Tissue Samples

First, 33 pairs of BC tissue and normal breast tissue were obtained from the Second Affiliated Hospital of Xi'an Jiaotong University (Xi'an, China). Next, BC tissues of 27 other patients were collected. Specimens were put into RNAlater solution, immediately after surgery, and stored at −80°C. In addition, 3-mm-thick tumor tissues from 10 patients were fixed in formalin and then embedded in paraffin. All the patients were diagnosed with primary non-metastatic BC by pathology and did not receive chemotherapy or radiotherapy before surgery. The clinicopathological features were collected from patients, and each patient gave informed consent. The study was approved by the Ethics Committee of Xi'an Jiaotong University.

RNA Isolation and qRT-PCR

Total RNA was extracted from tissue samples using TRIzol reagent (Invitrogen, Waltham, MA, USA) and was reverse transcribed into cDNA using the PrimeScript RT Reagent Kit (TaKaRa, Tokyo, Japan) following the manufacturer's protocol. The expression levels of *MNX1* were determined by qRT-PCR using the SYBR Premix Ex Taq II Kit (TaKaRa, Tokyo, Japan) on the ABI StepOne Real-Time

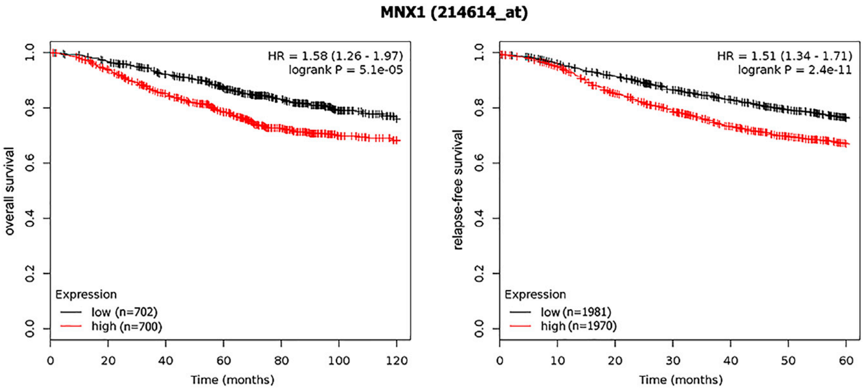

Figure 4. The Association between *MNX1* Expression and BC Survival
Patients with higher *MNX1* expression had a poor survival compared to patients with lower *MNX1* expression.

PCR system. GAPDH was used as an internal control. The primers are as follows: *MNX1* forward: 5′-CACTCGCGTGGGAGTTTG TG-3′; reverse: 5′-CCAATAATCAAAGTCGCCGCC-3′; GAPDH forward: 5′-GACAG TCAGCCGCATCTTCT-3′; reverse: 5′-GCGC CCAATACGACCAAATC-3′. The relative mRNA expression was calculated using the $2^{-\Delta\Delta Ct}$ method.

Immunohistochemistry

Tissue sections were deparaffinized with xylene and rehydrated with graded alcohols. After antigen retrieval, 3% hydrogen peroxide was used to block the endogenous peroxidase activity. Then, slices were blocked by 10% goat plasma and incubated with 0.1% Triton X-100. Subsequently, the sections were incubated with anti-MNX1 antibody (5 μg/mL, Abcam, Cambridge, MA, USA) at 4°C overnight. After incubation with biotinylated secondary antibody and horseradish-peroxidase-labeled streptavidin, the sections were detected by DAB and counterstained with hematoxylin. Images were captured on the Nikon Eclipse 50i microscope.

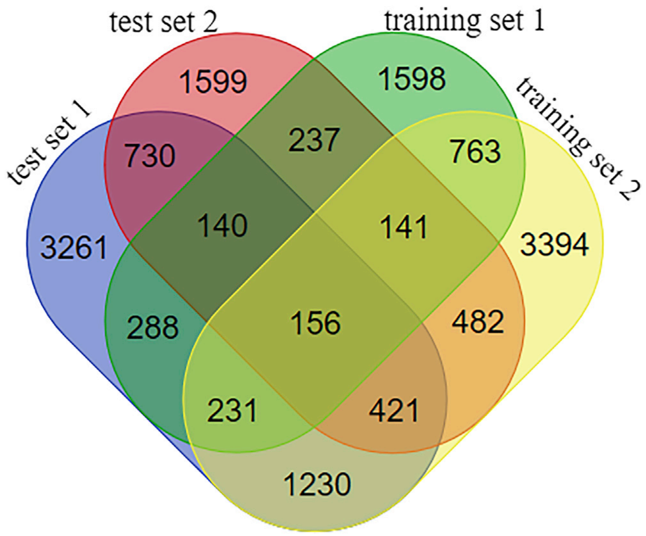

Figure 5. Venn Diagram of Overlapping Genes in the Four Gene Sets
The overlapped genes were identified as significant *MNX1*-related DEGs in BC.

Statistical Analysis

Statistical tests were carried out using IBM SPSS Statistics 22.0 software. The difference in *MNX1* expression between BC tissues and normal breast tissues was examined by Welch's t test. A chi-square test was applied to evaluate the association of *MNX1* expression with BC clinicopathological features.

Mining Correlated Genes

Prospective correlated genes of *MNX1* were analyzed through LinkedOmics using the Pearson correlation test. Genes with a p value < 0.01 and correlation coefficient (corrcoef) ≥ 0.1 were chosen to form the test set. In order to increase the credibility, we used the *MNX1*-related genes mined from the Multi Experiment Matrix (MEM) database (https://biit.cs.ut.ee/mem/) as another test set.[23] In addition, we mined DEGs in BC through GEPIA (http://gepia.cancer-pku.cn/).[24] Fold change (FC) ≥ 1 and p value < 0.01 were defined as the cutoff to select significant DEGs, which consist of a training set. We also used PALM-IST (http://www.hpppi.iicb.res.in/ctm/index.html) to mine BC-associated genes from the literature, which also formed a training set. The overlapping genes of the four sets were finally identified as significant *MNX1*-related DEGs in BC. The Venn diagram for overlapping genes was drawn by an online tool (http://bioinformatics.psb.ugent.be/webtools/Venn/).

Protein Interaction Analysis

The PPIs for the *MNX1* and its correlated genes were analyzed by STRING (v.10.5; http://www.string-db.org/).[25] A combined score of ≥0.4 was used as the cutoff for significant interaction. The PPI network was then visualized using the software Cytoscape (v.3.5.1). Module analysis was carried out by MCODE, with the criteria of a degree ≥2 and k-core ≥3. Hub genes were selected by cytoHubba.[26] As there are 12 topological algorithms, we used the median as the cutoff to find the overlapping genes according to their score under each algorithm, which were designated as hub genes.

Functional Enrichment Analysis

Functional enrichment analysis for *MNX1*-correlated genes along with *MNX1* was performed by Metascape (http://metascape.org).[27] The enrichment analysis comprises the GO biological process (BP), Kyoto Encyclopedia of Genes and Genomes (KEGG) pathways, and Reactome pathways. Only terms with p values < 0.01 and a number of enriched genes ≥3 were considered as significant. All the resultant

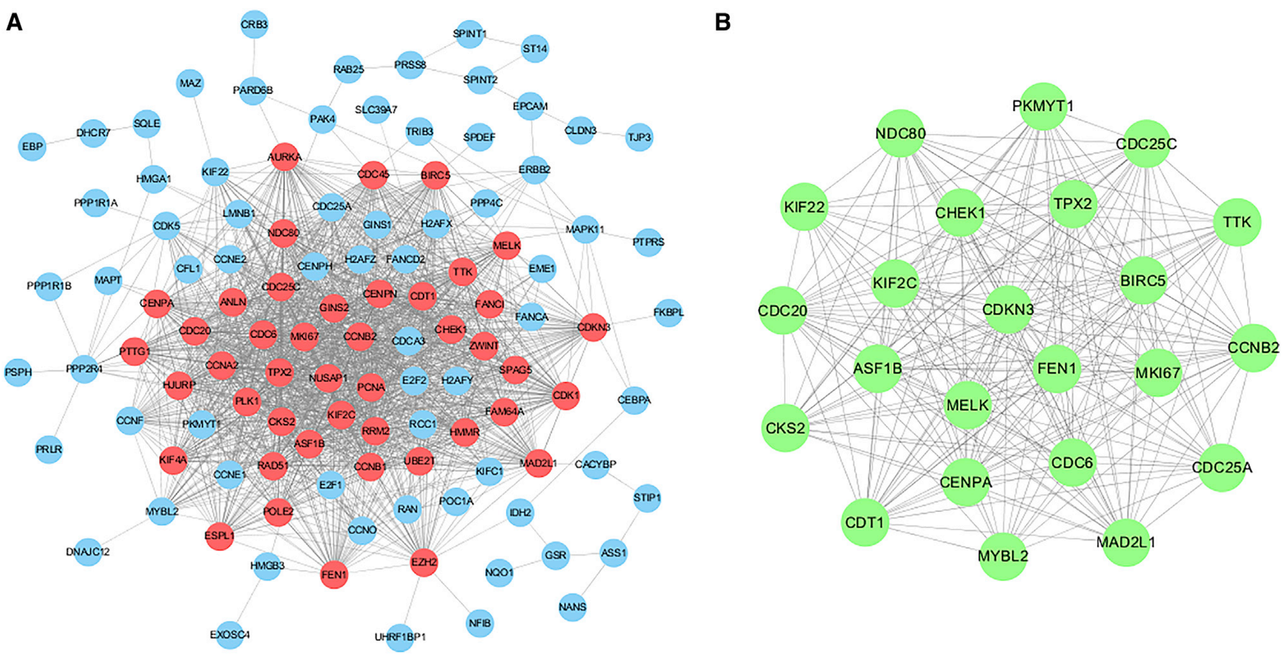

Figure 6. PPI Network of *MNX1*-Correlated Genes

(A) Whole network of the interacting proteins in BC. The pink nodes denote the most highly interconnected proteins in the network. (B) Sub-network of hub genes. Each protein was interacted with more than 10 other proteins in the network.

terms were then grouped into clusters, based on their similarities. The most enriched term within a cluster was chosen as the one to represent the cluster.

SUPPLEMENTAL INFORMATION

Supplemental Information includes two tables and can be found with this article online at https://doi.org/10.1016/j.omtn.2018.09.014.

AUTHOR CONTRIBUTIONS

T.T. and Z.-J.D. conceived and designed the study. T.T., M.W., and Y.Z. retrieved databases and collected data. S.L., C.D., and Y.D. organized and collated data. T.T. and T.Y. analyzed and interpreted data. T.T. and D.S. performed experiments. N.L. and Z.Z. prepared tables and figures. T.T. and M.W. drafted the manuscript. Z.-J.D., W.Z., and H.L. revised the manuscript. All authors approved the final manuscript.

CONFLICTS OF INTEREST

The authors have no conflicts of interest.

ACKNOWLEDGMENTS

This work was supported by the National Natural Science Foundation, People's Republic of China (no. 81471670); the Key Research and Development Plan, Shaanxi Province, People's Republic of China (2017ZDXM-SF-066); and the Science and Technology Branch

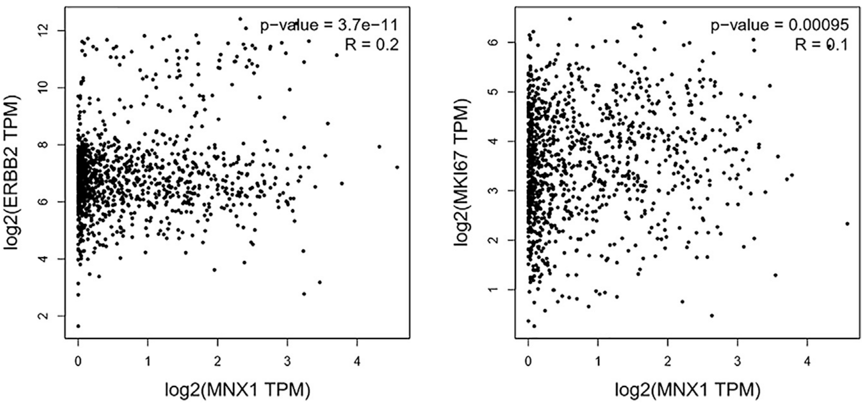

Figure 7. The Correlation of *MNX1* Expression with *ERBB2* (*HER2*) and *MKI67* Expression

Pearson correlation coefficients were calculated using data from TCGA.

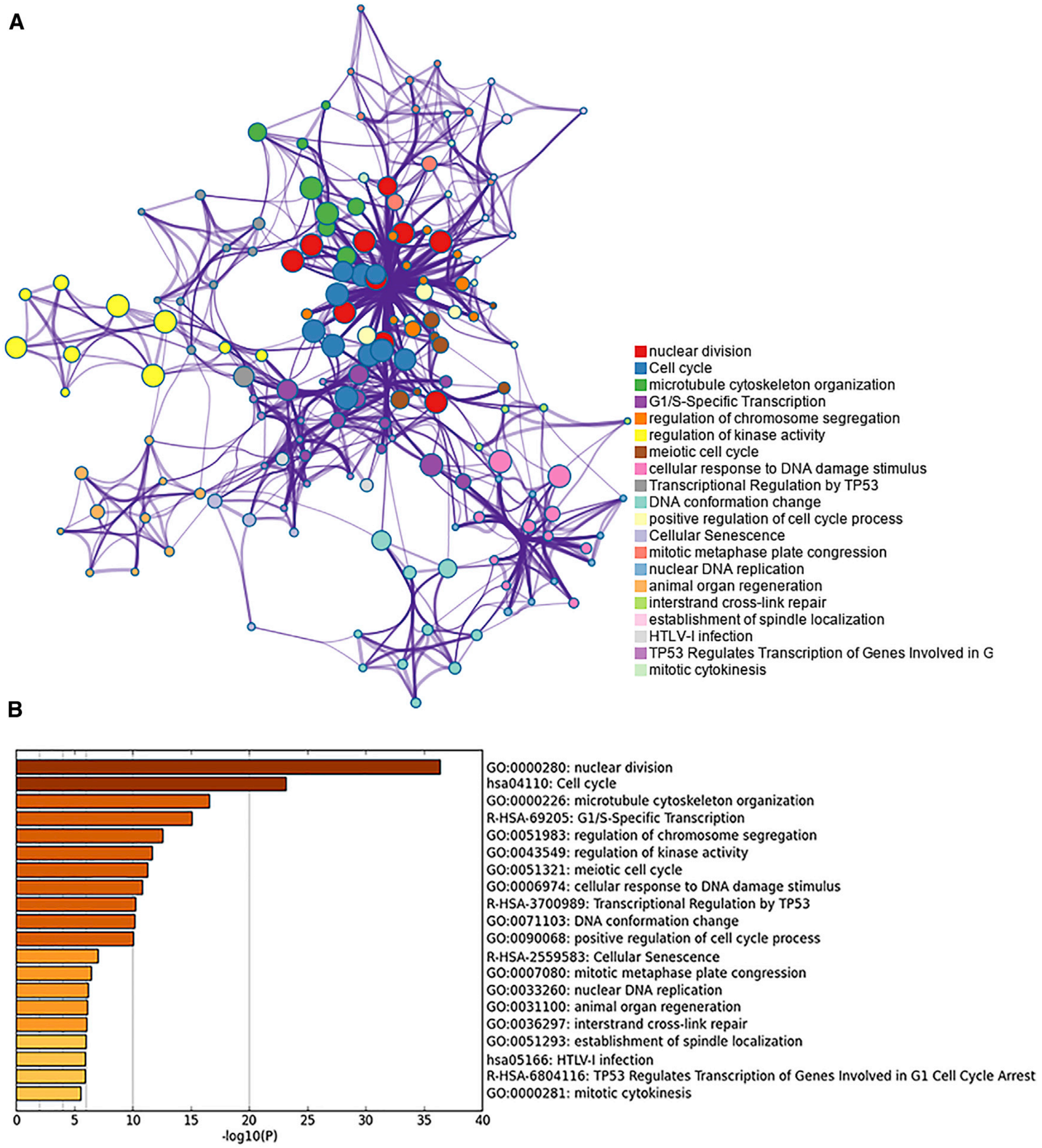

Figure 8. The 20 Top-Score Clusters of Significantly Enriched Terms

(A) Network of the 20 clusters comprising the 10 best terms within each. Each node represents one enriched term, and each color represents a cluster. Terms paired with kappa similarity above 0.3 were connected. The thicker the edge displayed, the higher the similarity is. (B) Heatmap of the 20 top-score clusters. Each bar represents a cluster. The darker the color of the bar is, the smaller the p value is.

Project of the Xinjiang Uygur Autonomous Region, People's Republic of China (2017E0262). The sponsor had no role in study design, data collection, data analysis, data interpretation, or writing of the report.

REFERENCES

1. Bray, F., Ferlay, J., Soerjomataram, I., Siegel, R.L., Torre, L.A., and Jemal, A. (2018). Global cancer statistics 2018: GLOBOCAN estimates of incidence and mortality worldwide for 36 cancers in 185 countries. CA Cancer J. Clin. Published online September 12, 2018. https://doi.org/10.3322/caac.21492.

2. Arnedos, M., Vicier, C., Loi, S., Lefebvre, C., Michiels, S., Bonnefoi, H., and Andre, F. (2015). Precision medicine for metastatic breast cancer–limitations and solutions. Nat. Rev. Clin. Oncol. *12*, 693–704.

3. Leotta, C.G., Federico, C., Brundo, M.V., Tosi, S., and Saccone, S. (2014). HLXB9 gene expression, and nuclear location during in vitro neuronal differentiation in the SK-N-BE neuroblastoma cell line. PLoS ONE *9*, e105481.

4. Vult von Steyern, F., Martinov, V., Rabben, I., Njå, A., de Lapeyrière, O., and Lømo, T. (1999). The homeodomain transcription factors Islet 1 and HB9 are expressed in adult alpha and gamma motoneurons identified by selective retrograde tracing. Eur. J. Neurosci. *11*, 2093–2102.

5. Harrison, K.A., Druey, K.M., Deguchi, Y., Tuscano, J.M., and Kehrl, J.H. (1994). A novel human homeobox gene distantly related to proboscipedia is expressed in lymphoid and pancreatic tissues. J. Biol. Chem. *269*, 19968–19975.

6. Li, H., Arber, S., Jessell, T.M., and Edlund, H. (1999). Selective agenesis of the dorsal pancreas in mice lacking homeobox gene Hlxb9. Nat. Genet. *23*, 67–70.

7. Ross, A.J., Ruiz-Perez, V., Wang, Y., Hagan, D.M., Scherer, S., Lynch, S.A., Lindsay, S., Custard, E., Belloni, E., Wilson, D.I., et al. (1998). A homeobox gene, HLXB9, is the major locus for dominantly inherited sacral agenesis. Nat. Genet. *20*, 358–361.

8. Belloni, E., Martucciello, G., Verderio, D., Ponti, E., Seri, M., Jasonni, V., Torre, M., Ferrari, M., Tsui, L.C., and Scherer, S.W. (2000). Involvement of the HLXB9 homeobox gene in Currarino syndrome. Am. J. Hum. Genet. *66*, 312–319.

9. Zhang, L., Wang, J., Wang, Y., Zhang, Y., Castro, P., Shao, L., Sreekumar, A., Putluri, N., Guha, N., Deepak, S., et al. (2016). MNX1 is oncogenically upregulated in African-American prostate cancer. Cancer Res. *76*, 6290–6298.

10. Wilkens, L., Jaggi, R., Hammer, C., Inderbitzin, D., Giger, O., and von Neuhoff, N. (2011). The homeobox gene HLXB9 is upregulated in a morphological subset of poorly differentiated hepatocellular carcinoma. Virchows Arch. *458*, 697–708.

11. von Bergh, A.R., van Drunen, E., van Wering, E.R., van Zutven, L.J., Hainmann, I., Lönnerholm, G., Meijerink, J.P., Pieters, R., and Beverloo, H.B. (2006). High incidence of t(7;12)(q36;p13) in infant AML but not in infant ALL, with a dismal outcome and ectopic expression of HLXB9. Genes Chromosomes Cancer *45*, 731–739.

12. Das, M. (2016). MNX1: a novel prostate cancer oncogene. Lancet Oncol. *17*, e521.

13. Desai, S.S., Kharade, S.S., Parekh, V.I., Iyer, S., and Agarwal, S.K. (2015). Pro-oncogenic roles of HLXB9 protein in insulinoma cells through interaction with Nono protein and down-regulation of the c-Met inhibitor Cblb (Casitas B-lineage Lymphoma b). J. Biol. Chem. *290*, 25595–25608.

14. Neufing, P.J., Kalionis, B., Horsfall, D.J., Ricciardelli, C., Stahl, J., Vivekanandan, S., Raymond, W., and Tilley, W.D. (2003). Expression and localization of homeodomain proteins DLX4/HB9 in normal and malignant human breast tissues. Anticancer Res *23*, 1479–1488.

15. Koch, L. (2017). Cancer genomics: The driving force of cancer evolution. Nat. Rev. Genet. *18*, 703.

16. Borad, M.J., and LoRusso, P.M. (2017). Twenty-first century precision medicine in oncology: genomic profiling in patients with cancer. Mayo Clin. Proc. *92*, 1583–1591.

17. Gray, J., and Druker, B. (2012). Genomics: the breast cancer landscape. Nature *486*, 328–329.

18. Nagel, S., Scherr, M., Quentmeier, H., Kaufmann, M., Zaborski, M., Drexler, H.G., and MacLeod, R.A. (2005). HLXB9 activates IL6 in Hodgkin lymphoma cell lines and is regulated by PI3K signalling involving E2F3. Leukemia *19*, 841–846.

19. Desai, S.S., Modali, S.D., Parekh, V.I., Kebebew, E., and Agarwal, S.K. (2014). GSK-3β protein phosphorylates and stabilizes HLXB9 protein in insulinoma cells to form a targetable mechanism of controlling insulinoma cell proliferation. J. Biol. Chem. *289*, 5386–5398.

20. Ferguson, S., Gautrey, H.E., and Strathdee, G. (2011). The dual role of HLXB9 in leukemia. Pediatr. Blood Cancer *56*, 349–352.

21. Vasaikar, S.V., Straub, P., Wang, J., and Zhang, B. (2018). LinkedOmics: analyzing multi-omics data within and across 32 cancer types. Nucleic Acids Res. *46* (D1), D956–D963.

22. Györffy, B., Lanczky, A., Eklund, A.C., Denkert, C., Budczies, J., Li, Q., and Szallasi, Z. (2010). An online survival analysis tool to rapidly assess the effect of 22,277 genes on breast cancer prognosis using microarray data of 1,809 patients. Breast Cancer Res. Treat. *123*, 725–731.

23. Adler, P., Kolde, R., Kull, M., Tkachenko, A., Peterson, H., Reimand, J., and Vilo, J. (2009). Mining for coexpression across hundreds of datasets using novel rank aggregation and visualization methods. Genome Biol. *10*, R139.

24. Tang, Z., Li, C., Kang, B., Gao, G., Li, C., and Zhang, Z. (2017). GEPIA: a web server for cancer and normal gene expression profiling and interactive analyses. Nucleic Acids Res. *45* (W1), W98–W102.

25. Szklarczyk, D., Morris, J.H., Cook, H., Kuhn, M., Wyder, S., Simonovic, M., Santos, A., Doncheva, N.T., Roth, A., Bork, P., et al. (2017). The STRING database in 2017: quality-controlled protein-protein association networks, made broadly accessible. Nucleic Acids Res. *45*, D362–D368.

26. Chin, C.H., Chen, S.H., Wu, H.H., Ho, C.W., Ko, M.T., and Lin, C.Y. (2014). cytoHubba: identifying hub objects and sub-networks from complex interactome. BMC Syst. Biol. *8* (*Suppl 4*), S11.

27. Tripathi, S., Pohl, M.O., Zhou, Y., Rodriguez-Frandsen, A., Wang, G., Stein, D.A., Moulton, H.M., DeJesus, P., Che, J., Mulder, L.C., et al. (2015). Meta- and orthogonal integration of influenza "OMICs" data defines a role for UBR4 in virus budding. Cell Host Microbe *18*, 723–735.

OMTN, Volume *13*

Supplemental Information

Expression, Clinical Significance, and Functional Prediction of *MNX1* in Breast Cancer

Tian Tian, Meng Wang, Yuyao Zhu, Wenge Zhu, Tielin Yang, Hongtao Li, Shuai Lin, Cong Dai, Yujiao Deng, Dingli Song, Na Li, Zhen Zhai, and Zhi-Jun Dai

Supplementary Table2. The enriched pathways

Category	Term	P-value	Count
Reactome	R-HSA-1640170: Cell Cycle	1.00E-31	43
Reactome	R-HSA-69278: Cell Cycle, Mitotic	1.00E-30	40
KEGG	hsa04110: Cell cycle	1.00E-23	21
Reactome	R-HSA-69205: G1/S-Specific Transcription	1.00E-15	9
Reactome	R-HSA-113510: E2F mediated regulation of DNA replication	1.00E-14	10
Reactome	R-HSA-1538133:G0 and Early G1	1.00E-13	9
Reactome	R-HSA-453279: Mitotic G1-G1/S phases	1.00E-13	15
Reactome	R-HSA-69273: Cyclin A/B1 associated events during G2/M transition	1.00E-12	8
Reactome	R-HSA-2500257: Resolution of Sister Chromatid Cohesion	1.00E-12	13
Reactome	R-HSA-69206: G1/S Transition	1.00E-12	13
KEGG	hsa04114: Oocyte meiosis	1.00E-12	13
Reactome	R-HSA-68877: Mitotic Prometaphase	1.00E-12	13
Reactome	R-HSA-68886: M Phase	1.00E-11	18
Reactome	R-HSA-69620: Cell Cycle Checkpoints	1.00E-11	15
Reactome	R-HSA-156711: Polo-like kinase mediated events	1.00E-11	7
Reactome	R-HSA-6791312: TP53 Regulates Transcription of Cell Cycle Genes	1.00E-11	9
Reactome	R-HSA-3700989: Transcriptional Regulation by TP53	1.00E-10	18
Reactome	R-HSA-453274: Mitotic G2-G2/M phases	1.00E-10	14
Reactome	R-HSA-69242: S Phase	1.00E-10	12
Reactome	R-HSA-69306: DNA Replication	2.00E-10	11
Reactome	R-HSA-69275: G2/M Transition	6.31E-10	13
Reactome	R-HSA-68882: Mitotic Anaphase	7.94E-10	13
Reactome	R-HSA-2555396: Mitotic Metaphase and Anaphase	7.94E-10	13
KEGG	hsa04914: Progesterone-mediated oocyte maturation	7.94E-10	10
Reactome	R-HSA-2467813: Separation of Sister Chromatids	5.01E-09	12
Reactome	R-HSA-69478: G2/M DNA replication checkpoint	1E-08	4
Reactome	R-HSA-157881: Cyclin B2 mediated events	1E-08	4
Reactome	R-HSA-5663220: RHO GTPases Activate Formins	2.00E-08	10
Reactome	R-HSA-195258: RHO GTPase Effectors	2.00E-08	14
Reactome	R-HSA-176417: Phosphorylation of Emi1	2.51E-08	4
Reactome	R-HSA-69239: Synthesis of DNA	3.16E-08	9
Reactome	R-HSA-73886: Chromosome Maintenance	5.01E-08	9
Reactome	R-HSA-2980767: Activation of NIMA Kinases NEK9, NEK6, NEK7	6.31E-08	4
Reactome	R-HSA-2559583: Cellular Senescence	1E-07	11
Reactome	R-HSA-6804114: TP53 Regulates Transcription of Genes Involved in G2 Cell Cycle Arrest	1.26E-07	5
Reactome	R-HSA-453276: Regulation of mitotic cell cycle	1.26E-07	8
Reactome	R-HSA-174143: APC/C-mediated degradation of cell cycle proteins	1.26E-07	8
Reactome	R-HSA-73894: DNA Repair	2.00E-07	13

Reactome	R-HSA-69481G: 2/M Checkpoints	2.51E-07	10
KEGG	**hsa04115: p53 signaling pathway**	3.98E-07	7
Reactome	R-HSA-5693538: Homology Directed Repair	3.98E-07	9
Reactome	R-HSA-75035: Chk1/Chk2(Cds1) mediated inactivation of Cyclin B:Cdk1 complex	7.94E-07	4
Reactome	R-HSA-176814: Activation of APC/C and APC/C:Cdc20 mediated degradation of mitotic proteins	0.000001	7
Reactome	R-HSA-2559586: DNA Damage/Telomere Stress Induced Senescence	0.000001	7
KEGG	hsa05166: HTLV-I infection	1.26E-06	11
Reactome	R-HSA-6804116: TP53 Regulates Transcription of Genes Involved in G1 Cell Cycle Arrest	1.26E-06	4
Reactome	**R-HSA-194315: Signaling by Rho GTPases**	1.58E-06	14
KEGG	hsa03460: Fanconi anemia pathway	1.58E-06	6
Reactome	R-HSA-162658: Golgi Cisternae Pericentriolar Stack Reorganization	1.58E-06	4
Reactome	R-HSA-5693532: DNA Double-Strand Break Repair	2.00E-06	9
Reactome	R-HSA-69190: DNA strand elongation	2.00E-06	5
Reactome	R-HSA-69298: Association of licensing factors with the pre-replicative complex	2.51E-06	4
Reactome	R-HSA-5693567: HDR through Homologous Recombination (HR) or Single Strand Annealing (SSA)	3.16E-06	8
Reactome	R-HSA-176187: Activation of ATR in response to replication stress	3.98E-06	5
KEGG	**hsa05206: MicroRNAs in cancer**	5.01E-06	11
Reactome	R-HSA-6783310: Fanconi Anemia Pathway	6.31E-06	5
Reactome	R-HSA-606279: Deposition of new CENPA-containing nucleosomes at the centromere	0.00001	6
Reactome	R-HSA-774815: Nucleosome assembly	0.00001	6
Reactome	R-HSA-176409: APC/C:Cdc20 mediated degradation of mitotic proteins	1.26E-05	6
Reactome	R-HSA-8863678: Neurodegenerative Diseases	1.58E-05	4
Reactome	R-HSA-8862803: Deregulated CDK5 triggers multiple neurodegenerative pathways in Alzheimer's disease models	1.58E-05	4
Reactome	R-HSA-176408: Regulation of APC/C activators between G1/S and early anaphase	2.00E-05	6
Reactome	R-HSA-2980766: Nuclear Envelope Breakdown	2.00E-05	5
Reactome	R-HSA-68874: M/G1 Transition	2.51E-05	6
Reactome	R-HSA-69002: DNA Replication Pre-Initiation	2.51E-05	6
Reactome	R-HSA-113507: E2F-enabled inhibition of pre-replication complex	3.16E-05	3
Reactome	**R-HSA-6804756: Regulation of TP53 Activity through Phosphorylation**	3.98E-05	6
Reactome	R-HSA-68875: Mitotic Prophase	3.98E-05	7
Reactome	R-HSA-68689: CDC6 association with the ORC:origin complex	5.01E-05	3

Reactome	R-HSA-2514853: Condensation of Prometaphase Chromosomes	5.01E-05	3
KEGG	hsa05161: Hepatitis B	5.01E-05	7
Reactome	R-HSA-2262752: Cellular responses to stress	5.01E-05	12
Reactome	R-HSA-68962: Activation of the pre-replicative complex	6.31E-05	4
Reactome	R-HSA-176974: Unwinding of DNA	6.31E-05	3
Reactome	R-HSA-69615: G1/S DNA Damage Checkpoints	0.0001	5
Reactome	R-HSA-5685942: HDR through Homologous Recombination (HRR)	0.0001	5
Reactome	**R-HSA-5633007: Regulation of TP53 Activity**	0.0001	7
Reactome	R-HSA-4419969: Depolymerisation of the Nuclear Lamina	0.000126	3
Reactome	R-HSA-8854518: AURKA Activation by TPX2	0.000126	5
Reactome	R-HSA-2299718: Condensation of Prophase Chromosomes	0.000126	5
Reactome	R-HSA-69304: Regulation of DNA replication	0.0002	5
Reactome	R-HSA-8852276: The role of GTSE1 in G2/M progression after G2 checkpoint	0.0002	5
Reactome	R-HSA-2559580: Oxidative Stress Induced Senescence	0.0002	6
Reactome	R-HSA-157579: Telomere Maintenance	0.000251	5
KEGG	**hsa05222: Small cell lung cancer**	0.000251	5
Reactome	R-HSA-176412: Phosphorylation of the APC/C	0.000251	3
KEGG	**hsa05215: Prostate cancer**	0.000316	5
KEGG	hsa00100: Steroid biosynthesis	0.000316	3
Reactome	R-HSA-2565942: Regulation of PLK1 Activity at G2/M Transition	0.000316	5
Reactome	R-HSA-5651801: PCNA-Dependent Long Patch Base Excision Repair	0.000316	3
KEGG	hsa01522: Endocrine resistance	0.000398	5
Reactome	R-HSA-69473: G2/M DNA damage checkpoint	0.000398	5
KEGG	hsa00480: Glutathione metabolism	0.000501	4
Reactome	R-HSA-174048: APC/C:Cdc20 mediated degradation of Cyclin B	0.000501	3
Reactome	R-HSA-110373: Resolution of AP sites via the multiple-nucleotide	0.000501	3
Reactome	R-HSA-191273: Cholesterol biosynthesis	0.000501	3
Reactome	R-HSA-174417: Telomere C-strand (Lagging Strand) Synthesis	0.000501	3
Reactome	R-HSA-983189: Kinesins	0.000631	4
KEGG	**hsa05212: Pancreatic cancer**	0.000794	4
KEGG	hsa04530: Tight junction	0.001	6
Reactome	R-HSA-180786: Extension of Telomeres	0.001	3
Reactome	R-HSA-420029: Tight junction interactions	0.001	3
Reactome	R-HSA-68867: Assembly of the pre-replicative complex	0.001259	4
KEGG	**hsa05200: Pathways in cancer**	0.001259	9
KEGG	hsa03410: Base excision repair	0.001259	3
Reactome	R-HSA-69656: Cyclin A:Cdk2-associated events at S phase entry	0.001259	4
Reactome	R-HSA-3301854: Nuclear Pore Complex (NPC) Disassembly	0.001585	3
Reactome	R-HSA-6920: Cyclin E associated events during G1/S transition	0.001585	4
Reactome	R-HSA-8856688: Golgi-to-ER retrograde transport	0.001585	5

Reactome	R-HSA-174184: Cdc20:Phospho-APC/C mediated degradation of Cyclin A	0.001585	4
Reactome	R-HSA-174178: APC/C:Cdh1 mediated degradation of Cdc20 and other APC/C:Cdh1 targeted proteins in late mitosis/earl	0.001585	4
Reactome	R-HSA-179419: APC:Cdc20 mediated degradation of cell cycle proteins prior to satisfation of the cell cycle checkpo	0.001585	4
KEGG	hsa03030: DNA replication	0.001585	3
Reactome	R-HSA-73884: Base Excision Repair	0.001995	3
Reactome	R-HSA-73933: Resolution of Abasic Sites (AP sites)	0.001995	3
KEGG	**hsa05203: Viral carcinogenesis**	0.001995	6
KEGG	**hsa05219: Bladder cancer**	0.002512	3
Reactome	R-HSA-4615885: SUMOylation of DNA replication proteins	0.003162	3
Reactome	R-HSA-6811434: COPI-dependent Golgi-to-ER retrograde traffic	0.003981	4
Reactome	R-HSA-5693607: Processing of DNA double-strand break ends	0.003981	4
KEGG	**hsa05223: Non-small cell lung cancer**	0.005012	3
Reactome	R-HSA-983231: Factors involved in megakaryocyte development and platelet production	0.00631	5
Reactome	R-HSA-1655829: Regulation of cholesterol biosynthesis by SREBP (SREBF)	0.00631	3
KEGG	hsa04360: Axon guidance	0.00631	5
Reactome	R-HSA-187577: SCF(Skp2)-mediated degradation of p27/p21	0.007943	3
Reactome	R-HSA-421270: Cell-cell junction organization	0.007943	3
Reactome	R-HSA-1500620: Meiosis	0.007943	4
Reactome	R-HSA-5334118: DNA methylation	0.01	3
Reactome	R-HSA-6811442: Intra-Golgi and retrograde Golgi-to-ER traffic	0.01	5
Reactome	R-HSA-69580: p53-Dependent G1/S DNA damage checkpoint	0.01	3
Reactome	R-HSA-69563: p53-Dependent G1 DNA Damage Response	0.01	3
